# Human Neutrophil Clearance of Bacterial Pathogens Triggers Anti-Microbial γδ T Cell Responses in Early Infection

**DOI:** 10.1371/journal.ppat.1002040

**Published:** 2011-05-12

**Authors:** Martin S. Davey, Chan-Yu Lin, Gareth W. Roberts, Sinéad Heuston, Amanda C. Brown, James A. Chess, Mark A. Toleman, Cormac G. M. Gahan, Colin Hill, Tanya Parish, John D. Williams, Simon J. Davies, David W. Johnson, Nicholas Topley, Bernhard Moser, Matthias Eberl

**Affiliations:** 1 Department of Infection, Immunity and Biochemistry, School of Medicine, Cardiff University, Cardiff, United Kingdom; 2 Department of Nephrology, Chang Gung Memorial Hospital, Taoyuan, Taiwan; 3 Institute of Nephrology, School of Medicine, Cardiff University, Cardiff, United Kingdom; 4 Alimentary Pharmabiotic Centre and Department of Microbiology, University College Cork, Cork, Ireland; 5 Centre for Immunology and Infectious Disease, Queen Mary University of London, Barts and The London School of Medicine and Dentistry, London, United Kingdom; 6 Department of Nephrology, Morriston Hospital, Swansea, United Kingdom; 7 Department of Nephrology, University Hospital of North Staffordshire, Keele University, Stoke-on-Trent, United Kingdom; 8 Department of Nephrology, Princess Alexandra Hospital, University of Queensland, Brisbane, Australia; 9 Australia and New Zealand Dialysis Transplant Registry, University of Adelaide, Adelaide, Australia; University of Pennsylvania, United States of America

## Abstract

Human blood Vγ9/Vδ2 T cells, monocytes and neutrophils share a responsiveness toward inflammatory chemokines and are rapidly recruited to sites of infection. Studying their interaction *in vitro* and relating these findings to *in vivo* observations in patients may therefore provide crucial insight into inflammatory events. Our present data demonstrate that Vγ9/Vδ2 T cells provide potent survival signals resulting in neutrophil activation and the release of the neutrophil chemoattractant CXCL8 (IL-8). In turn, Vγ9/Vδ2 T cells readily respond to neutrophils harboring phagocytosed bacteria, as evidenced by expression of CD69, interferon (IFN)-γ and tumor necrosis factor (TNF)-α. This response is dependent on the ability of these bacteria to produce the microbial metabolite (*E*)-4-hydroxy-3-methyl-but-2-enyl pyrophosphate (HMB-PP), requires cell-cell contact of Vγ9/Vδ2 T cells with accessory monocytes through lymphocyte function-associated antigen-1 (LFA-1), and results in a TNF-α dependent proliferation of Vγ9/Vδ2 T cells. The antibiotic fosmidomycin, which targets the HMB-PP biosynthesis pathway, not only has a direct antibacterial effect on most HMB-PP producing bacteria but also possesses rapid anti-inflammatory properties by inhibiting γδ T cell responses *in vitro*. Patients with acute peritoneal-dialysis (PD)-associated bacterial peritonitis – characterized by an excessive influx of neutrophils and monocytes into the peritoneal cavity – show a selective activation of local Vγ9/Vδ2 T cells by HMB-PP producing but not by HMB-PP deficient bacterial pathogens. The γδ T cell-driven perpetuation of inflammatory responses during acute peritonitis is associated with elevated peritoneal levels of γδ T cells and TNF-α and detrimental clinical outcomes in infections caused by HMB-PP positive microorganisms. Taken together, our findings indicate a direct link between invading pathogens, neutrophils, monocytes and microbe-responsive γδ T cells in early infection and suggest novel diagnostic and therapeutic approaches.

## Author Summary

The immune system of all jawed vertebrates harbors three distinct lymphocyte populations – αβ T cells, γδ T cells and B cells – yet only higher primates including humans possess so-called Vγ9/Vδ2 T cells, an enigmatic γδ T cell subset that uniformly responds to the majority of bacterial pathogens. For reasons that are not understood, this responsiveness is absent in all other animals although they too are constantly exposed to a plethora of potentially harmful micro-organisms. We here investigated how Vγ9/Vδ2 T cells respond to live microbes by mimicking physiological conditions in acute disease. Our experiments demonstrate that Vγ9/Vδ2 T cells recognize a small common molecule released when invading bacteria become ingested and killed by other white blood cells. The stimulation of Vγ9/Vδ2 T cells at the site of infection amplifies the inflammatory response and has important consequences for pathogen clearance and the development of microbe-specific immunity. However, if triggered at the wrong time or the wrong place, this rapid reaction toward bacteria may also lead to inflammation-related damage. These findings improve our insight into the complex cellular interactions in early infection, identify novel biomarkers of diagnostic and predictive value and highlight new avenues for therapeutic intervention.

## Introduction

The cellular immune system consists of an ‘innate’ arm of phagocytes and antigen-presenting cells, and an ‘adaptive’ arm of antigen-specific lymphocytes capable of developing immunological memory. Yet, there is increasing evidence of considerable crosstalk between the two [1]. Innate responses directly influence the shape and outcome of adaptive T cell responses, and *vice versa* specialized T cell subsets feedback to innate cells [2]. Among these interactions, the regulation of neutrophil-mediated inflammatory responses by Th17 cells has received enormous attention over the past few years [3], and with the emergence of novel T cell subsets additional networks are being proposed so that each polarized T cell eventually pairs with an innate counter player [4]–[7].

The necessity to integrate complex signals in order to mount the most effective defense is best illustrated by the existence of ‘unconventional’ T cells bridging the classical divide between innate and adaptive immunity, such as natural killer T cells, mucosal-associated invariant T cells, intestinal intraepithelial CD8αα^+^ T cells and dendritic epidermal γδ T cells [8]–[14]. These often tissue-associated lymphocytes are characterised by restricted T cell receptor (TCR) repertoires that allow them to respond rapidly to a limited range of conserved structures. Unconventional T cells readily assume a plethora of effector functions, ranging from sentinel tasks and targeted killing to engaging with keratinocytes, fibroblasts, phagocytes and antigen-presenting cells as well as other lymphocyte.

γδ T cells expressing a Vγ9/Vδ2 TCR – Vγ2/Vδ2 according to an alternative nomenclature – are only found in humans and higher primates and differ fundamentally from all other conventional and unconventional T cells [15]. Activated Vγ9/Vδ2 T cells produce a range of cytokines, kill infected and transformed target cells, regulate survival and differentiation of monocytes and maturation of dendritic cells, provide B cell help and present antigens to CD4^+^ and CD8^+^ T cells [11], [12], [16], [17]. They expand considerably in many infections, at times to >50% of all circulating T cells within a few days [18], and respond selectively in a non-MHC restricted manner to the microbial metabolite (*E*)-4-hydroxy-3-methyl-but-2-enyl pyrophosphate (HMB-PP) [19]. HMB-PP is an intermediate of the non-mevalonate pathway of isoprenoid biosynthesis that is present in many bacteria and in malaria parasites but not in humans [17]–[19]. The rapid and sensitive response of Vγ9/Vδ2 T cells to a broad range of pathogens evokes cardinal features of innate immunity. Indeed, HMB-PP fulfills Janeway's criteria for a ‘pathogen-associated molecular pattern’ in that it is (*i*) invariant among different species; (*ii*) a product of a pathway unique to micro-organisms; and (*iii*) essential in microbial physiology [17]. Yet, HMB-PP recognition is not mediated *via* germline-encoded pattern recognition receptors but involves the re-arranged Vγ9/Vδ2 TCR [20]–[22].

Bacteria that possess the non-mevalonate pathway and hence produce HMB-PP comprise some of the most detrimental human pathogens such as the causative agents of cholera, diphtheria, plague, tuberculosis and typhoid, but also numerous commensal and opportunistic species in the mucosal flora, skin and feces [19], [23]. In all these micro-organisms, HMB-PP is an essential intracellular metabolite, and it is not clear whether and how it is released by invading bacteria and becomes visible to the immune system as soluble molecule. Indeed, earlier studies with mycobacteria suggested that uptake of whole bacteria by monocytes, macrophages, or DCs may be required for the recognition by Vγ9/Vδ2 T cells [24]–[27]. Neutrophils are the first immune cells infiltrating the site of infection and the main phagocytes responsible for early pathogen clearance, and growing evidence points toward a crucial role of γδ T cells in regulating neutrophil responses in mouse models of infection, hypersensitivity and autoimmunity [8], [12]. Yet, the interplay between γδ T cells and neutrophils has not been addressed in detail [28], [29]. Our present data demonstrate that Vγ9/Vδ2 T cells readily respond to neutrophils harboring phagocytosed bacteria, and that this response is strictly dependent on the ability of these bacteria to produce HMB-PP and cell-cell contact of Vγ9/Vδ2 T cells with accessory monocytes. The majority of circulating Vγ9/Vδ2 T cells shows migration properties similar to monocytes [30], suggesting that these two cell types are co-recruited to the site of inflammation and interact with each other at early stages of infection [17], [31]. Our present findings thus indicate a direct link between invading pathogens, neutrophils, monocytes and microbe-responsive γδ T cells, and suggest novel diagnostic and therapeutic approaches in acute infection.

## Results

### Human γδ T cells induce neutrophil survival and activation

**Figure 1:**
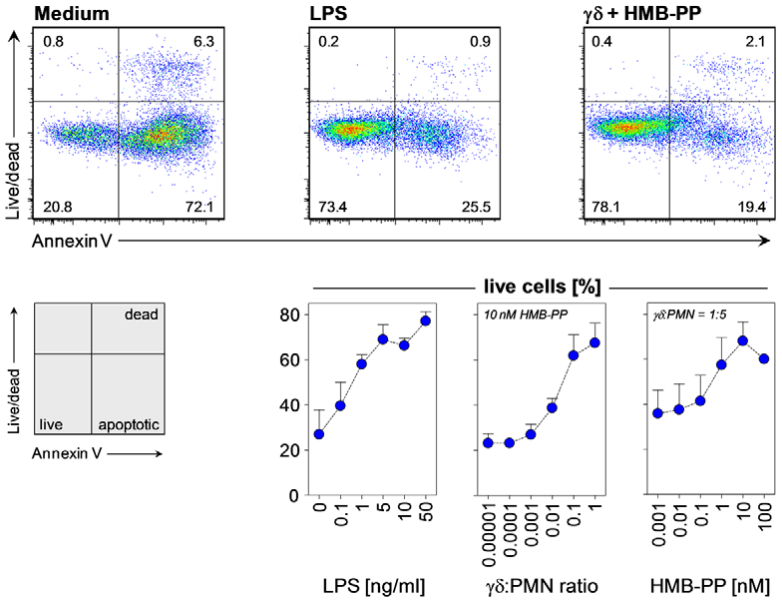
HMB-PP stimulated Vγ9/Vδ2 T cells induce neutrophil survival. Dose-dependent survival of neutrophils incubated with LPS, and of neutrophils (PMN) co-cultured with Vγ9/Vδ2 T cells at various ratios in the presence of HMB-PP at different concentrations. Neutrophils were analyzed after 20 hours in culture; dot plots depict representative annexin-V and fixable live/dead stainings for live CD3^−^ CD15^+^ neutrophils cultured under the indicated conditions. Data shown are mean percentages (%) + SEM of live cells from independent experiments using three different donors.

Neutrophils are short-lived phagocytes that undergo spontaneous apoptosis *in vitro* unless rescued by survival signals. We previously demonstrated that activated human Vγ9/Vδ2 T cells induce monocytes to survive and differentiate into inflammatory dendritic cells [31]. Here, HMB-PP stimulated Vγ9/Vδ2 T cells had a similar survival effect on autologous neutrophils and readily rescued them from undergoing apoptosis (Figure 1). This effect was selective and dependent on the number of Vγ9/Vδ2 T cells and the concentration of HMB-PP. An increase in neutrophil survival could already be observed at ratios of only 1 γδ T cell per 100 neutrophils and at HMB-PP concentrations as low as 0.1–1 nM. Activation of Vγ9/Vδ2 T cells in these cultures was confirmed by upregulation of CD69 and secretion of interferon (IFN)-γ (Figure S1 in Text S1). The low γδ T cell numbers and HMB-PP concentrations needed to promote neutrophil survival *in vitro* are likely to have physiologic relevance.

**Figure 2:**
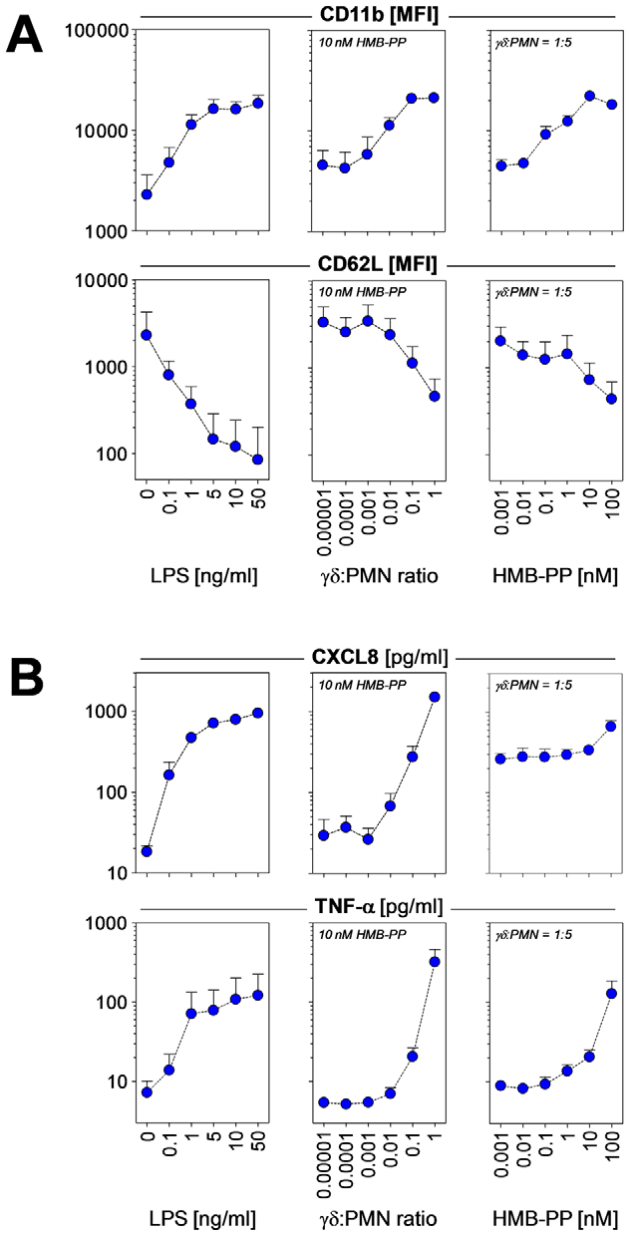
HMB-PP stimulated Vγ9/Vδ2 T cells induce neutrophil activation and production of pro-inflammatory mediators. (***A***) Dose-dependent up-regulation of CD11b and shedding of CD62L by neutrophils incubated with LPS as positive control, and of neutrophils co-cultured with Vγ9/Vδ2 T cells at various ratios in the presence of HMB-PP at different concentrations. Neutrophils were analyzed after 20 hours in culture; histograms depict representative CD11b and CD62L stainings for live CD3^−^ CD15^+^ neutrophils cultured under the indicated conditions. Data shown are mean fluorescence intensities (MFI) + SEM from independent experiments using three different donors. (***B***) Dose-dependent secretion of CXCL8 and TNF-α into the culture supernatant of neutrophils incubated with LPS as positive control, and of neutrophils co-cultured with Vγ9/Vδ2 T cells at various ratios in the presence of HMB-PP at different concentrations. Supernatants were analyzed after 20 hours by ELISA. Data shown are mean levels (pg/ml) + SEM from independent experiments using three different donors.

Activated neutrophils mobilize intracellular stores of CD11b to the cell surface and shed CD62L, thus enhancing their potential to undergo firm adhesions with endothelial cells and extravasate at the site of inflammation. In line with their anti-apoptotic effect on neutrophils, Vγ9/Vδ2 T cells induced upregulation of CD11b and loss of CD62L in surviving neutrophils in an HMB-PP dependent manner (Figure 2A). Importantly, synthetic HMB-PP alone did not have any activity on neutrophils in the absence of γδ T cells (Figure 1, Figure 2 and data not shown).

**Figure 3:**
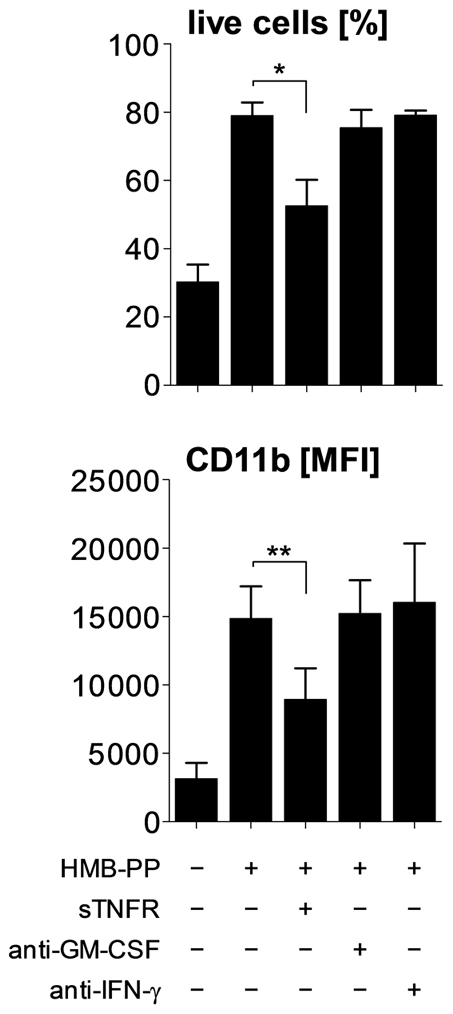
HMB-PP stimulated Vγ9/Vδ2 T cells induce neutrophil survival and activation through TNF-α. Neutrophils were co-cultured with Vγ9/Vδ2 T cells for 20 hours at a ratio of 5∶1, in the absence or presence of 10 nM HMB-PP. Soluble cytokines were blocked by the addition of sTNFR, anti-GM-CSF or anti-IFN-γ. Data shown are mean values + SEM for neutrophil survival and expression of CD11b by live neutrophils, as determined in independent experiments using three different donors.

Rapid recruitment of neutrophils involves the chemotactic action of CXCL8 (IL-8) produced at the site of inflammation, and increased endothelial permeability mediated by tumor necrosis factor (TNF)-α. Analysis of the supernatants from the above experiments revealed that co-cultures of neutrophils and HMB-PP stimulated Vγ9/Vδ2 T cells produced considerable amounts of both CXCL8 and TNF-α, in a dose-dependent manner and at levels comparable to lipopolysaccharide (LPS) stimulated neutrophils (Figure 2B). Another cytokine implicated in neutrophil recruitment is IL-17, which in a number of infection models is readily produced by murine γδ T cells [8]. While activated Vγ9/Vδ2 T cells readily produce TNF-α, IFN-γ and granulocyte/macrophage colony-stimulating factor (GM-CSF) [31], [32], we were unable to detect IL-17 in our co-cultures indicating that under the conditions tested human γδ T cells failed to secrete relevant levels of IL-17 (data not shown). This is reminiscent of recent findings that human αβ T cells including human Th17 cells modulate neutrophils (which lack the IL-17 receptor C chain) in an IL-17–independent manner through a combination of TNF-α, IFN-γ and GM-CSF [33]. In our cultures, blocking experiments demonstrated that TNF-α played a key role in the γδ T cell-mediated effect on neutrophils, as judged by a partial inhibition of neutrophil survival and a reduction of CD11b expression in the presence of soluble TNF-α receptor (sTNFR), while neutralizing antibodies against GM-CSF and IFN-γ had no significant effect (Figure 3).

Taken together, these data show that Vγ9/Vδ2 T cells become activated by soluble HMB-PP in the presence of autologous neutrophils and that they provide potent stimulatory signals inducing neutrophil survival and activation. The interaction of the two cell types leads to the rapid release of the pro-inflammatory mediators CXCL8 and TNF-α into the microenvironment, thereby potentially maintaining neutrophil influx at the site of infection.

### Vγ9/Vδ2 T cells respond to neutrophils harboring phagocytosed bacteria

Under physiological conditions, invading pathogens are rapidly taken up by newly recruited neutrophils. We therefore tested whether Vγ9/Vδ2 T cells respond to neutrophils harboring phagocytosed bacteria in a similar manner as they respond to soluble HMB-PP. In order to do this, we set up triple cultures consisting of neutrophils, monocytes and Vγ9/Vδ2 T cells, mimicking physiological conditions at the site of infection.

**Figure 4:**
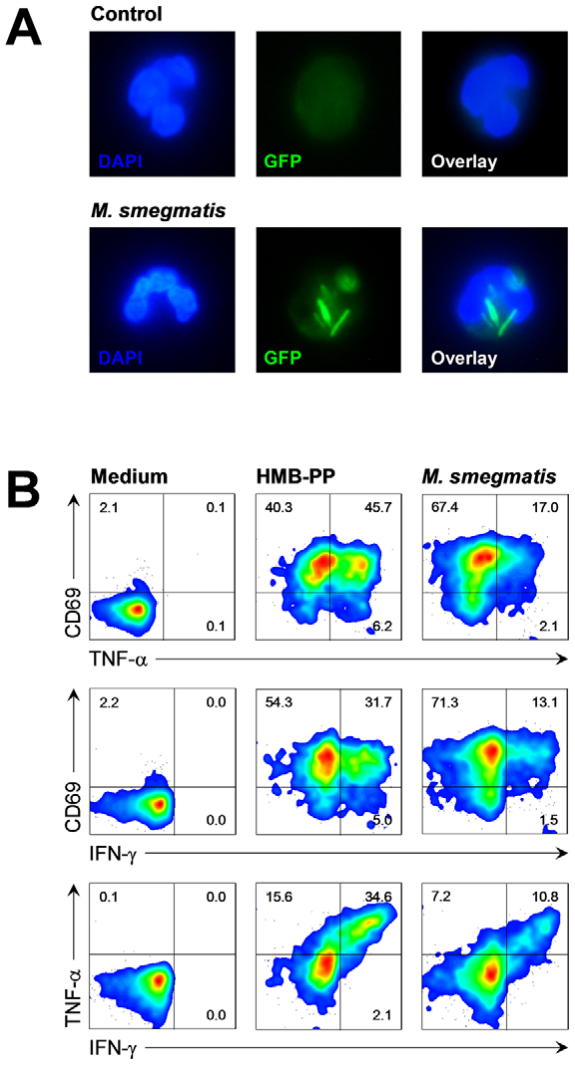
Vγ9/Vδ2 T cells respond to bacteria upon phagocytosis by neutrophils. (***A***) Resting neutrophils or neutrophils after phagocytosis of *M. smegmatis*-*gfp* ^+^ stably expressing GFP, at a multiplicity of infection (MOI) of 10. Cells were counter-stained with DAPI and imaged by fluorescence microscopy. Data shown are representative from independent experiments using two different donors. (***B***) Activation of Vγ9/Vδ2 T cells co-cultured for 20 hours with neutrophils in the absence (medium) or in the presence of 10 nM HMB-PP, or co-cultured with neutrophils harboring *M. smegmatis*-*gcpE* ^+^ overexpressing HMB-PP synthase. Data shown are representative from independent experiments using three different donors.

Human neutrophils readily took up green fluorescent protein (GFP) expressing *Escherichia coli*, *Listeria innocua* and *Mycobacterium smegmatis*, with >95% of the neutrophils being GFP^+^ within 30 min (Figure 4A and data not shown). Triple co-cultures of neutrophils harboring different strains of *M. smegmatis* with autologous Vγ9/Vδ2 T cells and monocytes led to rapid γδ T cell activation, as evidenced by upregulation of CD69 and expression of TNF-α and IFN-γ within 20 hours (Figure 4B and data not shown). Activation profiles were similar to those seen in control cultures with non-infected neutrophils in the presence of synthetic HMB-PP, demonstrating that Vγ9/Vδ2 T cells respond to bacterial degradation products released or presented by neutrophils. For the sake of clarity and simplicity all activation data in the following sections are shown as proportion of CD69^+^ TNF-α^+^ γδ T cells in the cultures although the cells were always co-stained for IFN-γ as well. The proportion of CD69^+^ IFN-γ^+^ and TNF-α^+^ IFN-γ^+^ γδ T cells followed essentially the same pattern throughout this study and led to the same conclusions.

### The Vγ9/Vδ2 T cell response to phagocytosed bacteria depends on the ability of bacteria to produce HMB-PP

In order to investigate the correlation between the ability of bacteria to produce HMB-PP and their capacity to stimulate Vγ9/Vδ2 T cells, we designed experiments to distinguish a specific γδ T cell response to HMB-PP from a possible background stimulation by the plethora of other microbial compounds acting *via* pattern recognition receptors. Thus, we generated a *M. smegmatis* transfectant stably expressing a second copy of the gene encoding HMB-PP synthase (*gcpE*) and hence overproducing HMB-PP compared to the parental wildtype (wt) strain [34] (Figure S2 in Text S1). As a second bacterial model we utilized HMB-PP producing and HMB-PP deficient strains of the non-pathogenic Gram-positive bacterium *Listeria innocua* [35], [36] (Figure S3 in Text S1).

**Figure 5:**
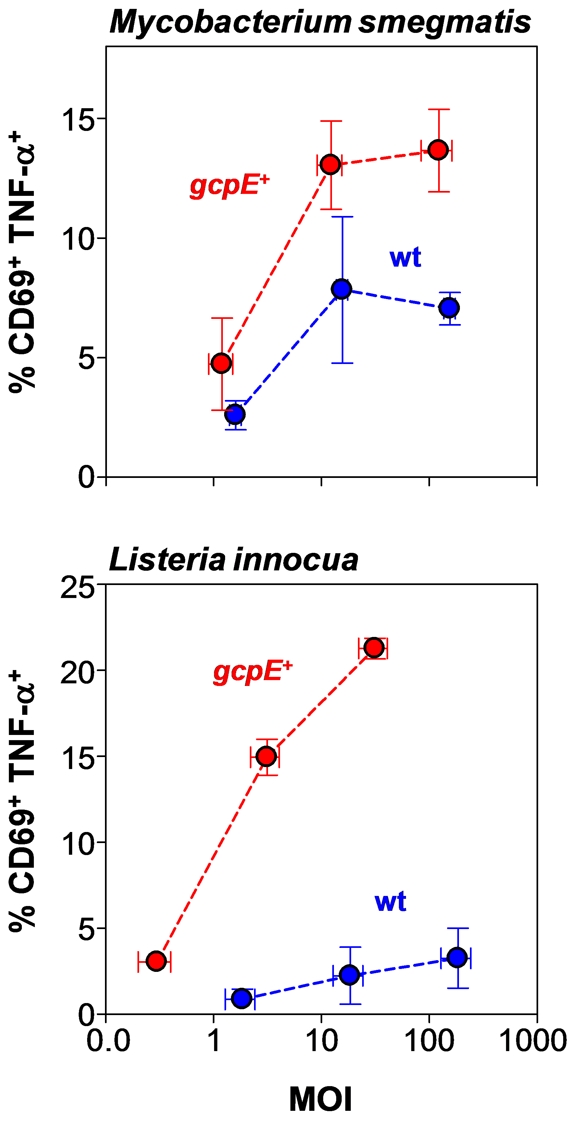
Vγ9/Vδ2 T cells show enhanced responses to phagocytosed bacteria that overproduce HMB-PP. Dose-dependent activation of Vγ9/Vδ2 T cells by neutrophils harboring genetically engineered transfectants of *M. smegmatis* (upper panel) or *L. innocua* (lower panel), in which HMB-PP accumulates intracellularly, compared to the parental wildtype strains (wt). Data shown are mean frequencies of CD69^+^ TNF-α^+^ Vγ9/Vδ2 T cells ± SEM after 20 hours in culture, as determined in independent experiments using three different donors. Error bars for MOIs depict mean values ± SEM for the true colony-forming unit (CFU) counts of the bacterial inocula used.

Compared with *M. smegmatis* wt bacteria, higher levels of Vγ9/Vδ2 T cell activation were observed when using *M. smegmatis*-*gcpE* ^+^ (Figure 5). Furthermore, considerable Vγ9/Vδ2 T cell activation was seen with phagocytosed *L. innocua*-*gcpE* ^+^, a strain in which HMB-PP artificially accumulates, but not with the naturally HMB-PP deficient *L. innocua* wt strain that was >100× less potent (Figure 5). The double transfectant *L. innocua*-*gcpE* ^+^ *lytB* ^+^, in which HMB-PP becomes converted into the downstream reaction products isopentenyl pyrophosphate (IPP) and dimethylallyl pyrophosphate (DMAPP), resulted in no detectable Vγ9/Vδ2 T cell activation (data not shown). These data demonstrate that the response of Vγ9/Vδ2 T cells to neutrophils harboring phagocytosed bacteria depends on the ability of these bacteria to produce HMB-PP and suggest that phagocytosis and subsequent degradation of bacteria in neutrophils leads to either presentation of HMB-PP on the cell surface or the release of soluble HMB-PP into the microenvironment.

### Crosstalk with monocytes provides essential help for the Vγ9/Vδ2 T cell response to phagocytosed bacteria

**Figure 6:**
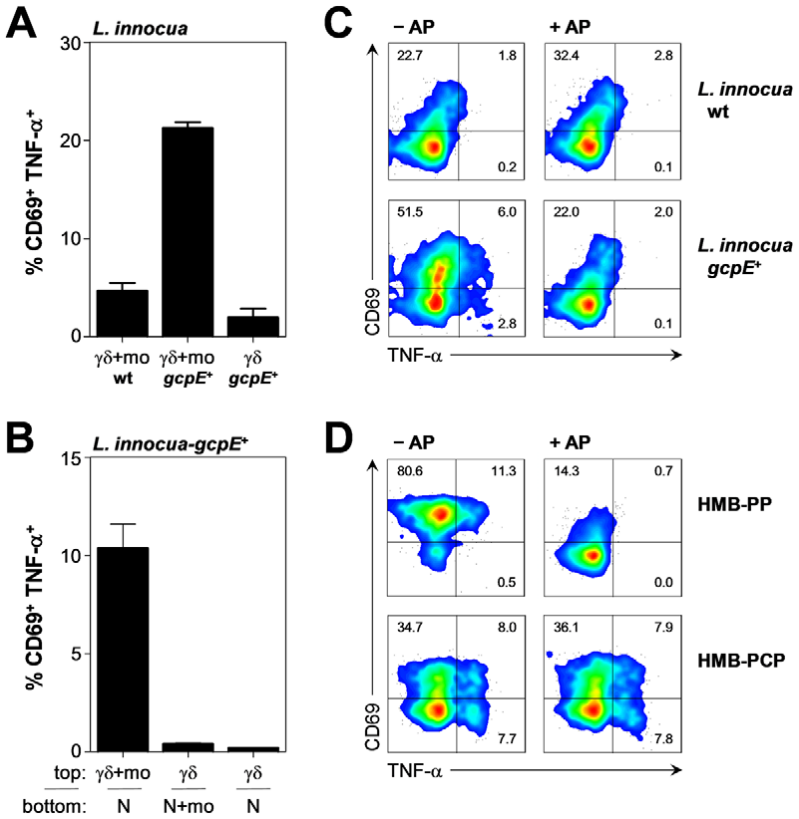
Vγ9/Vδ2 T cells respond, in a monocyte-dependent manner, to phosphatase-sensitive molecules released from phagocytosed bacteria. (***A***) Activation of Vγ9/Vδ2 T cells by neutrophils harboring *L. innocua* wt or *L. innocua*-*gcpE* ^+^ bacteria, in the presence (γδ-mo) or absence (γδ) of monocytes. Data shown are mean frequencies of CD69^+^ TNF-α^+^ Vγ9/Vδ2 T cells + SEM after 20 hours in culture, as determined in independent experiments using three different donors. (***B***) Activation of Vγ9/Vδ2 T cells in the top chamber of a transwell plate separated from neutrophils (N) harboring *L. innocua*-*gcpE* ^+^ in the bottom chamber, in the presence or absence of monocytes (mo). Data shown are mean frequencies of CD69^+^ TNF-α^+^ Vγ9/Vδ2 T cells + SEM after 20 hours in culture, as determined in independent experiments using three different donors. (***C***) Activation of Vγ9/Vδ2 T cells co-cultured for 20 hours with monocytes in the presence of supernatants from neutrophils harboring *L. innocua* wt or *L. innocua*-*gcpE* ^+^ bacteria that had been pretreated or not with alkaline phosphatase (AP). Data shown are representative from independent experiments using two different donors. (***D***) Activation of Vγ9/Vδ2 T cells co-cultured for 20 hours with monocytes in the presence of 1 nM HMB-PP or 100 µM HMB-PCP pretreated or not with alkaline phosphatase (AP). Data shown are representative from independent experiments using two different donors.

Vγ9/Vδ2 T cells, monocytes and neutrophils share a responsiveness toward inflammatory chemokines and are the earliest leukocytes recruited to sites of infection. Vγ9/Vδ2 T cell responses *in vitro* are greatly facilitated by contact with monocytes as ‘feeder cells’, which most likely act by ‘presenting’ HMB-PP to Vγ9/Vδ2 T cells and by providing contact-dependent signals [17]. In support of our previous observation of a substantial HMB-PP dependent crosstalk between Vγ9/Vδ2 T cells and monocytes leading to optimum γδ T cell activation [31], the response of Vγ9/Vδ2 T cells to neutrophils harboring phagocytosed *L. innocua*-*gcpE* ^+^ was largely dependent on the presence of monocytes. Omission of monocytes from the co-cultures resulted in greatly reduced expression levels of CD69, TNF-α and IFN-γ, compared to triple co-cultures (Figure 6A and data not shown), suggesting that monocytes provide essential help for the recognition of bacteria by Vγ9/Vδ2 T cells and increase the sensitivity of the response especially at low HMB-PP concentrations.

We speculated that this accessory effect might have stemmed from contact-dependent interactions of monocytes with either neutrophils or γδ T cells and tested this hypothesis in transwell cultures where neutrophils harboring phagocytosed *L. innocua*-*gcpE* ^+^ in the lower chamber were separated from Vγ9/Vδ2 T cells in the upper chamber. As shown in Figure 6B, cell-cell contact between monocytes and Vγ9/Vδ2 T cells was crucial for the response to phagocytosed bacteria, while no contact was needed between Vγ9/Vδ2 T cells and neutrophils, and neither between monocytes and neutrophils. These data indicate that upon phagocytosis of HMB-PP^+^ bacteria, neutrophils release soluble factors that efficiently stimulate Vγ9/Vδ2 T cells, while monocytes provide important contact-dependent accessory signals.

### Upon phagocytosis of HMB-PP^+^ bacteria neutrophils release HMB-PP into the culture supernatant

Since neutrophils harboring bacteria were able to stimulate Vγ9/Vδ2 T cells in a transwell system, we next examined whether cell-free culture supernatants derived from infected neutrophils stimulated Vγ9/Vδ2 T cells in a similar manner. Indeed, Vγ9/Vδ2 T cells readily responded to supernatants from neutrophils harboring *L. innocua*-*gcpE^+^* but not from neutrophils harboring *L. innocua* wt bacteria, as evidenced by expression of CD69, TNF-α and IFN-γ (Figure 6C and data not shown). Importantly, short-term pre-treatment of *L. innocua*-*gcpE^+^* supernatants with shrimp alkaline phosphatase abrogated the bioactivity on Vγ9/Vδ2 T cells completely (Figure 6C), evoking the known sensitivity of mycobacterial HMB-PP to dephosphorylation and the relative inactivity of the dephosphorylated products [37]–[39]. Control experiments confirmed that alkaline phosphatase affected the response of Vγ9/Vδ2 T cells to synthetic HMB-PP but not to the phosphatase-resistant diphosphonate analogue, HMB-PCP [40], demonstrating that the presence of alkaline phosphatase in the cultures had no inhibitory effect on the cells' ability to express CD69, TNF-α and IFN-γ (Figure 6D and data not shown). We conclude that upon phagocytosis of HMB-PP^+^ bacteria neutrophils release soluble HMB-PP into the microenvironment where it becomes accessible to monocytes and Vγ9/Vδ2 T cells.

### The Vγ9/Vδ2 T cell response to phagocytosed bacteria is HMB-PP dependent but largely independent of other pathogen-associated molecular patterns

**Figure 7:**
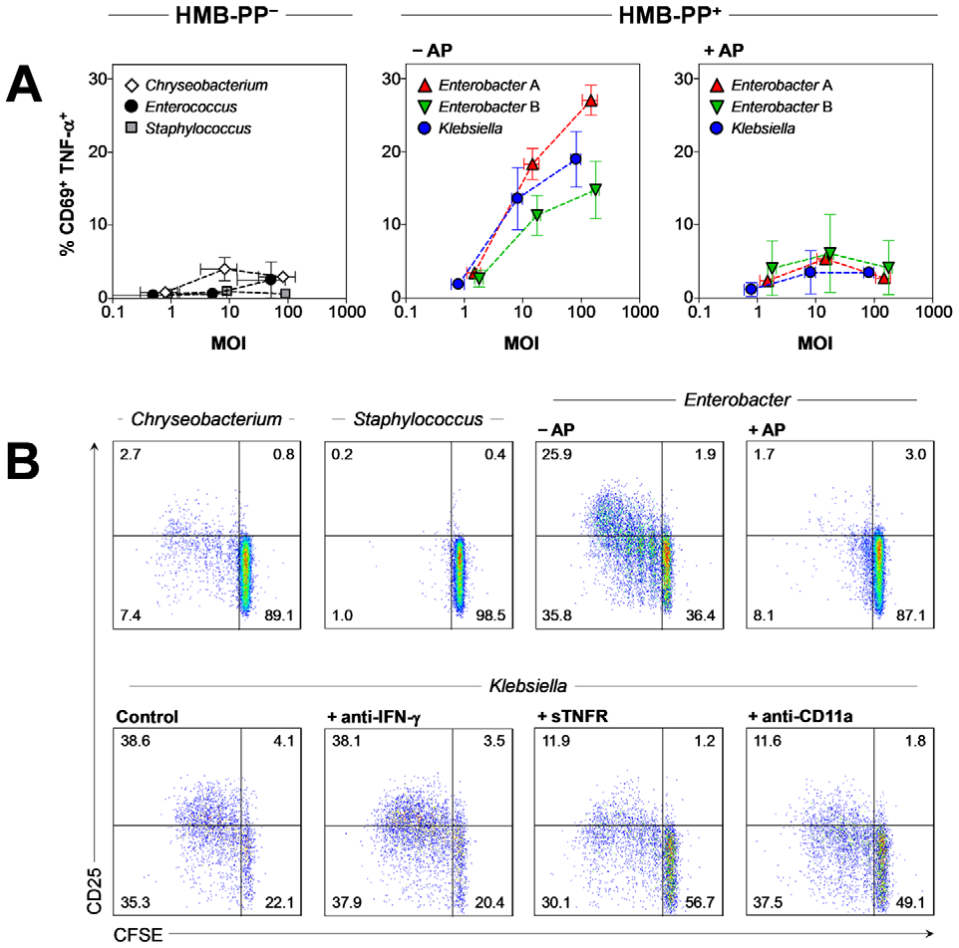
Vγ9/Vδ2 T cells respond to HMB-PP producing Gram^**+**^ and Gram^**−**^ bacteria but not to HMB-PP deficient bacteria. (***A***) Dose-dependent activation of Vγ9/Vδ2 T cells by neutrophils harboring clinical isolates of a range of different bacteria: Gram^−^ HMB-PP^+^, *Enterobacter cloacae* (two different isolates tested, A and B) and *Klebsiella pneumoniae*; Gram^−^ HMB-PP^−^, *Chryseobacterium indologenes*; and Gram^+^ HMB-PP^−^, *Enterococcus faecalis* and *Staphylococcus aureus*; in the presence or absence of alkaline phosphatase (AP). Data shown are mean frequencies of CD69^+^ TNF-α^+^ Vγ9/Vδ2 T cells ± SEM after 20 hours in culture, as determined in independent experiments using 3–5 donors. Error bars for MOIs depict mean values ± SEM for the true CFU counts of the bacterial inocula used. (***B***) Proliferation after 5 days in culture and CD25 expression of CFSE-labeled Vγ9/Vδ2 T cells in response to supernatants from neutrophils harboring clinical isolates of the indicated bacteria, in the presence or absence of alkaline phosphatase (AP), soluble TNF-α receptor (sTNFR) or blocking antibodies against IFN-γ or CD11a. Data shown are representative of independent experiments using cells from at least two different donors.

In order to assess the clinical relevance of our findings, we expanded our panel of bacteria by including clinical isolates of pathogens that are frequently associated with community- and hospital-acquired infections and pose serious threats to public health (Table S1 in Text S1). Of note, neutrophils harboring HMB-PP^+^ pathogens but not neutrophils harboring HMB-PP^−^ pathogens induced in Vγ9/Vδ2 T cells the co-expression of CD69, TNF-α and IFN-γ (Figure 7A and data not shown). This response was largely independent of the presence of other pathogen-associated molecular patterns such as LPS as both Gram-negative (*Acinetobacter baumannii*, *Enterobacter cloacae*, *Klebsiella pneumoniae*, *Pseudomonas aeruginosa*) and Gram-positive bacteria (*M. smegmatis*) capable of producing HMB-PP stimulated Vγ9/Vδ2 T cells equally. Direct addition of alkaline phosphatase to these co-cultures abrogated the HMB-PP dependent cytokine responses, confirming soluble HMB-PP as common Vγ9/Vδ2 T cell stimulator in these species (Figure 7A, Figure S4 in Text S1). The bioactivity of culture supernatants harvested after 5 hours from neutrophils harboring the above bacteria corresponded to levels of 0.1–10 nM HMB-PP, as titrated against a HMB-PP standard (data not shown). Residual levels of CD69 expression after phosphatase treatment may have been due to incomplete degradation of HMB-PP and to indirect stimulation of Vγ9/Vδ2 T cells by other microbial compounds such as LPS acting on neutrophils or monocytes [41], [42]. In contrast to HMB-PP producing species, HMB-PP deficient Gram-negative (*Chryseobacterium indologenes*) and Gram-positive bacteria (*Enterococcus faecalis*, *L. innocua*, *Staphylococcus aureus*) did not elicit Vγ9/Vδ2 T cells responses above background as demonstrated by the complete lack of TNF-α (Figure 7A, Figure S4 in Text S1) and IFN-γ (data not shown). These findings illustrate the extraordinary specificity Vγ9/Vδ2 T cells for HMB-PP, even in the abundant presence of other microbial products and despite high levels of monocyte and/or neutrophil-derived mediators such as IL-1β, IL-6 and CXCL8 that were present in our triple co-cultures regardless of the HMB-PP status of the phagocytosed bacteria (data not shown).

### Vγ9/Vδ2 T cells proliferate in response to phagocytosed bacteria in a TCR, LFA-1 and TNF-α dependent manner

γδ T cells expand rapidly in acute bacterial infections [18]. We therefore tested whether phagocytosed pathogens could induce expansion of 5-(and 6-)carboxyfluorescein diacetate succinimidyl ester (CFSE)-labeled Vγ9/Vδ2 T cells. As shown in Figure 7B, Vγ9/Vδ2 T cells proliferated considerably in the presence of supernatants derived from neutrophils harboring HMB-PP^+^ *Enterobacter cloacae* but not from neutrophils harboring HMB-PP^−^ *Chryseobacterium indologenes* or *Staphylococcus aureus*. Similarly to the immediate up-regulation of CD69, TNF-α and IFN-γ, the proliferation of Vγ9/Vδ2 T cells in response to *Enterobacter cloacae* was HMB-PP dependent and could be abrogated by alkaline phosphatase. Expanding Vγ9/Vδ2 T cells also up-regulated the high affinity IL-2 receptor, CD25 (Figure 7B) and became responsive to exogenously added IL-2, which enhanced the proliferative response even further (data not shown). Blocking experiments demonstrated a crucial requirement of soluble and contact-dependent signals for optimum stimulation of Vγ9/Vδ2 T cells. TNF-α was recently implicated in Vγ9/Vδ2 T cell proliferation in response to IPP and IL-2 [43], and blocking of lymphocyte function-associated antigen-1 (LFA-1, CD11a/CD18) efficiently disrupted cluster formation with monocytes [31]. Here, both Vγ9/Vδ2 T cell proliferation and CD25 up-regulation in response to supernatants derived from neutrophils harboring HMB-PP^+^ *Klebsiella pneumoniae* (Figure 7B) or *Enterobacter cloacae* (data not shown) were readily inhibited by sTNFR and anti-CD11a antibodies but not by anti-IFN-γ antibodies. Finally, addition of anti-Vγ9 antibodies completely abrogated the Vγ9/Vδ2 T cell proliferation in response to HMB-PP (data not shown) and *Enterobacter cloacae* supernatants (Figure S7 in Text S1), confirming a requirement for the TCR [44]. Taken together, our findings demonstrate that Vγ9/Vδ2 T cells are rapidly activated by a broad range of HMB-PP producing pathogens, leading to TCR, LFA-1 and TNF-α dependent γδ T cell expansion.

### Acute infections caused by HMB-PP producing bacteria are characterized by elevated numbers of activated γδ T cells

**Figure 8:**
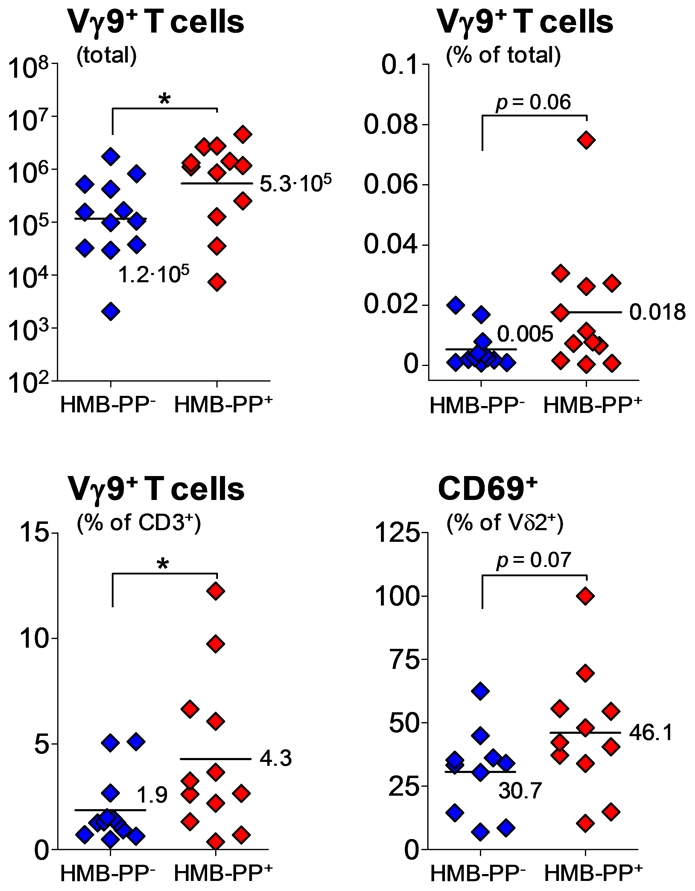
Vγ9/Vδ2 T cell numbers and CD69 expression are elevated in acute peritonitis caused by HMB-PP bacteria. Total numbers and frequencies of Vγ9/Vδ2 T cells (% of total cells and % of all CD3^+^ T cells), and expression of CD69 on peritoneal Vγ9/Vδ2 T cells in patients with PD-associated peritonitis on day 1 (the day of hospital admission with a cloudy effluent, *i.e.* before commencement of antibiotic therapy), depending on whether or not the causative pathogen was capable of producing HMB-PP.

We next addressed whether the dichotomy between HMB-PP^+^ and HMB-PP^−^ pathogens in their potential to trigger γδ T cells *in vitro* is replicated under physiological conditions *in vivo*. As clinical correlate for HMB-PP^+^ and HMB-PP^−^ infections, we analyzed episodes of acute bacterial infections in peritoneal dialysis (PD) patients, in whom the peritoneal catheter affords continuous and non-invasive access to the inflammatory infiltrate (Table S2 in Text S1). PD-associated peritonitis is characterized by a considerable influx of neutrophils and monocytes into the peritoneal cavity [45], [46], where the two cell types may become targets for local or infiltrating γδ T cells [31], [47]. Here, in a total of 24 newly recruited patients examined on the first day of acute peritonitis (*i.e.* before administration of antibiotics), both the total number and the frequency of peritoneal Vγ9/Vδ2 T cells were elevated in HMB-PP^+^ infections compared to HMB-PP^−^ infections, suggesting increased recruitment and/or proliferation in response to HMB-PP released by bacteria (Figure 8). Moreover, local activation was evidenced by higher percentages of Vγ9/Vδ2 T cells expressing CD69 in the HMB-PP^+^ patient group. In contrast, we did not see any significant differences in the numbers and frequencies of peritoneal neutrophils, monocytes/macrophages and total CD3^+^ T cells, regardless of the HMB-PP status of the causative pathogen (Figure S5 in Text S1). Similarly, while the proportion of Vγ9/Vδ2 T cells within peritoneal CD3^+^ T cells was clearly elevated in HMB-PP^+^ infections, CD4^+^ and CD8^+^ T cells showed no such bias (Figure S6 in Text S1).

### Episodes of peritonitis caused by HMB-PP producing bacteria are associated with poor clinical outcome

**Figure 9:**
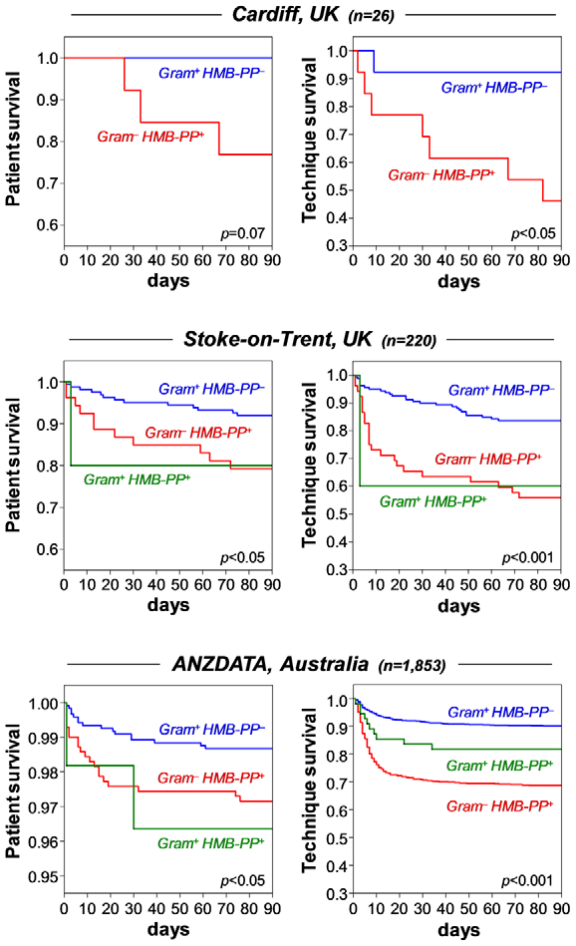
Episodes of peritonitis caused by HMB-PP producing bacteria are associated with poor clinical outcome. Cumulative patient survival (*left*) and cumulative technique survival (*right*) of patients with acute bacterial peritonitis, grouped into infections with Gram^+^ HMB-PP^−^ (blue), Gram^+^ HMB-PP^+^ (green) or Gram^−^ HMB-PP^+^ pathogens (red); episodes caused by Gram^−^ HMB-PP^−^ pathogens were not recorded in the patient databases. *Top*, PD patients admitted at the University Hospital of Wales, Cardiff, with acute peritonitis (day 1, *i.e.* first presentation with a cloudy bag). *Middle*, Australian PD patients from the ANZDATA registry with first-time peritonitis. *Bottom*, PD patients with first-time peritonitis treated at the University Hospital of North Staffordshire, Stoke-on-Trent, UK. Comparisons were made using log-rank tests.

As Medzhitov stated recently, “inflammation is beneficial in appropriate amounts but can easily become detrimental when excessive because of its tissue-damaging potential” [48]. PD patients constitute a particularly vulnerable group where inflammatory events can have profound consequences [49]–[51]. We speculated that local activation of γδ T cells may contribute to inflammation-related damage and tested whether the occurrence of clinical complications in PD patients depends on the capacity of the causative pathogen to produce HMB-PP. Our analysis of 26 patients treated at the University Hospital of Wales, Cardiff, UK, demonstrated that infections with HMB-PP^+^ bacteria were associated with worse outcomes, evidenced as higher mortality rates and higher incidences of technique failure (*i.e.*, cessation of therapy due to catheter removal, transfer to hemodialysis or patient death), while HMB-PP^−^ bacteria caused milder disease (Figure 9). Of note, we were able to validate this pattern in two larger and entirely independent cohorts treated in Australia (ANZDATA Registry) and at the University Hospital of North Staffordshire, Stoke-on-Trent, UK (Figure 9). In order to rule out that this pattern was not due to differences in Gram staining (and hence endotoxin-related), we divided the group of HMB-PP^+^ bacteria further into Gram^+^ and Gram^−^ species. Our outcome analysis demonstrates that even within the Gram^+^ group, bacteria capable of producing HMB-PP were associated with worse outcomes compared to HMB-PP^−^ pathogens (Figure 9), suggesting that the HMB-PP producing capacity of the causative pathogen might be of predictive value for the clinical outcome from bacterial peritonitis (Table S3 in Text S1).

### Peritoneal γδ T cell frequencies and TNF-α levels might predict clinical outcome in peritonitis patients

In order to identify potentially useful diagnostic and prognostic biomarkers of inflammation severity and outcomes from bacterial infection, we measured a large number of immunological parameters. These analyses identified elevated frequencies of peritoneal Vγ9/Vδ2 T cells on day 1 as possible predictor of subsequent technique failure within three months after infection (Table 1). Similarly, expression of the activation marker HLA-DR by peritoneal Vγ9/Vδ2 T cells on the day of admission was associated with increased mortality. No other parameters tested including the numbers and frequencies of neutrophils, monocytes or CD4^+^ and CD8^+^ T cells reached statistical significance (data not shown). Among soluble factors in peritoneal effluent, only elevated levels of TNF-α on day 1 indicated higher rates of technique failure and mortality (Table 1), while no such correlation was seen for other cytokines and chemokines, including GM-CSF, IFN-γ, IL-1β, IL-2, IL-6, IL-10, IL-12p70, IL-17, IL-22, CXCL8, CXCL10 and sIL-6R (data not shown).

**Table 1:** Identification of immune markers of possible predictive value for clinical outcome in 8 stable PD patients and 29 PD patients with acute peritonitis (means ± SD).

*90^th^ day technique failure*	Stable patients	Survivors	Non-survivors
Vγ9^+^ (% of CD3^+^ T cells)	1.5±0.9	2.5±2.1	4.9±3.2^*^
TNF-α (pg/ml)	3.1±4.3	41.9±53.3	128.4±91.0^*^
***90**^**th**^**day mortality***			
HLA-DR^+^ (% of Vδ2^+^ T cells)	14.7±10.5	11.5±10.5	40.6±7.5^***^
TNF-α (pg/ml)	3.1±4.3	48.1±60.4	148.7±90.2^*^

Asterisks indicate significant differences between survivors and non-survivors.

### Pre-treatment of bacteria with fosmidomycin blocks HMB-PP production and abrogates γδ T cell responses

Our findings of a rapid γδ T cell response to neutrophil-engulfed HMB-PP producing pathogens and its potential detrimental consequence in episodes of acute peritonitis may not only be of diagnostic and predictive value for affected patients, they also highlight possible new avenues of therapeutic intervention in bacterial infections. HMB-PP is an intermediate of the non-mevalonate pathway of isoprenoid biosynthesis, in which the first enzymatic step catalyzed by 1-deoxy-d-xylulose-5-phosphate reductoisomerase (Dxr) can be inhibited by fosmidomycin (Figure S8A in Text S1), a natural antibiotic produced by *Streptomyces lavendulae* [52], [53]. We therefore speculated that the effect of fosmidomycin pre-treatment of bacteria may serve a dual purpose in treating acute infections: by directly inhibiting an essential pathway in a broad range of pathogens and by abrogating HMB-PP driven inflammatory responses.

Tests with selected clinical isolates of common pathogens demonstrated that the majority of HMB-PP^+^ bacteria (*Enterobacter cloacae*, *Klebsiella pneumoniae*, *Pseudomonas aeruginosa*) was susceptible to overnight treatment with fosmidomycin (with the exception of *Acinetobacter baumannii* as expected [54]), with a mean inhibitory concentration (MIC) of 1–32 µg/ml depending on the strain (Table S1 in Text S1). Of note, fosmidomycin also acted on multidrug-resistant strains including bacteria harboring the recently discovered ‘New Delhi’ metallo-β-lactamase 1 (NDM-1) [55], [56] (Davey MS, Tyrrell JM *et al.*, submitted for publication). In contrast to the efficient killing of most HMB-PP^+^ bacteria, the HMB-PP^−^ bacteria *Chryseobacterium indologenes*, *Enterococcus faecalis* and *Staphylococcus aureus* were not affected by fosmidomycin (Table S1 in Text S1).

**Figure 10:**
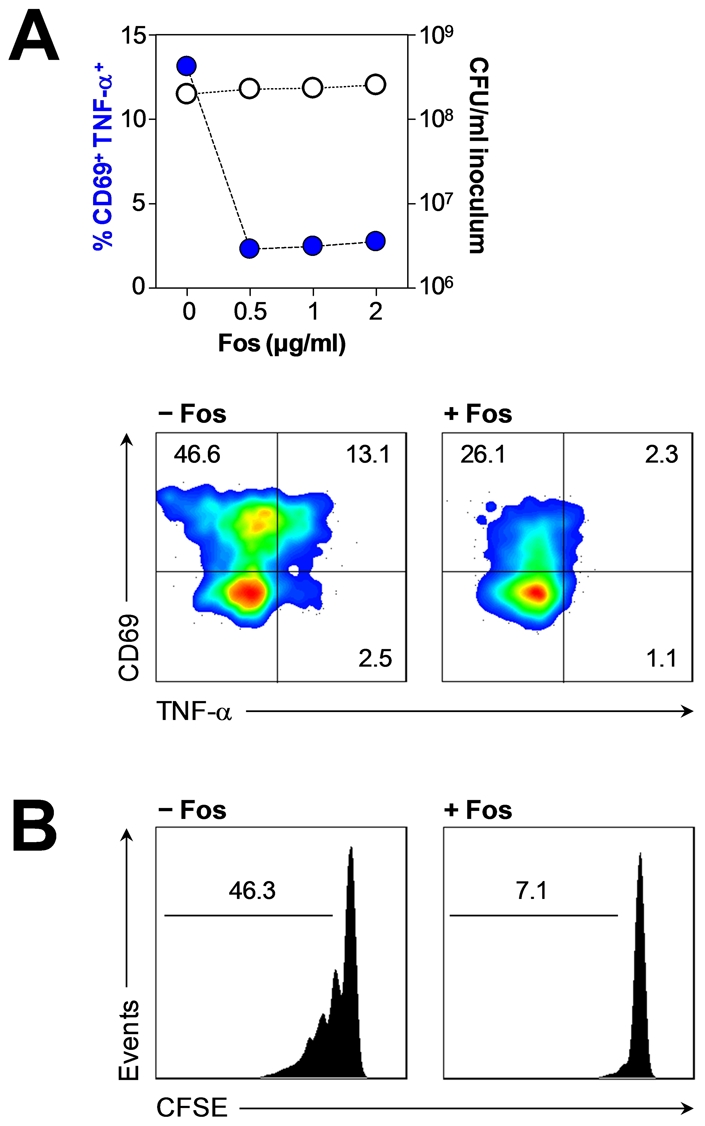
Abrogation of HMB-PP dependent γδ T cell responses by fosmidomycin. (***A***) Activation of Vγ9/Vδ2 T cells co-cultured for 20 hours with neutrophils harboring *Enterobacter cloacae* pre-treated or not for 1 hour with the indicated concentrations of fosmidomycin. The minimum inhibitory concentration (MIC) of fosmidomycin for this bacteria strain was 1 µg/ml, as determined by microbroth dilution (Table S1 in Text S1). Data shown are frequencies of CD69^+^ TNF-α^+^ Vγ9/Vδ2 T cells after 20 hours in culture, alongside CFU counts of the original bacterial inocula after 1 hour treatment with fosmidomycin, representative of independent experiments using cells from two different donors. FACS plots show typical Vγ9/Vδ2 T cell responses, representative of independent experiments using cells from three different donors. (***B***) Proliferation after 5 days in culture of CFSE-labeled Vγ9/Vδ2 T cells in response to supernatants from neutrophils harboring *Enterobacter cloacae* pre-treated or not for 1 hour with 0.25 µg/ml fosmidomycin. Data shown are representative of independent experiments using cells from two different donors.

**Figure 11:**
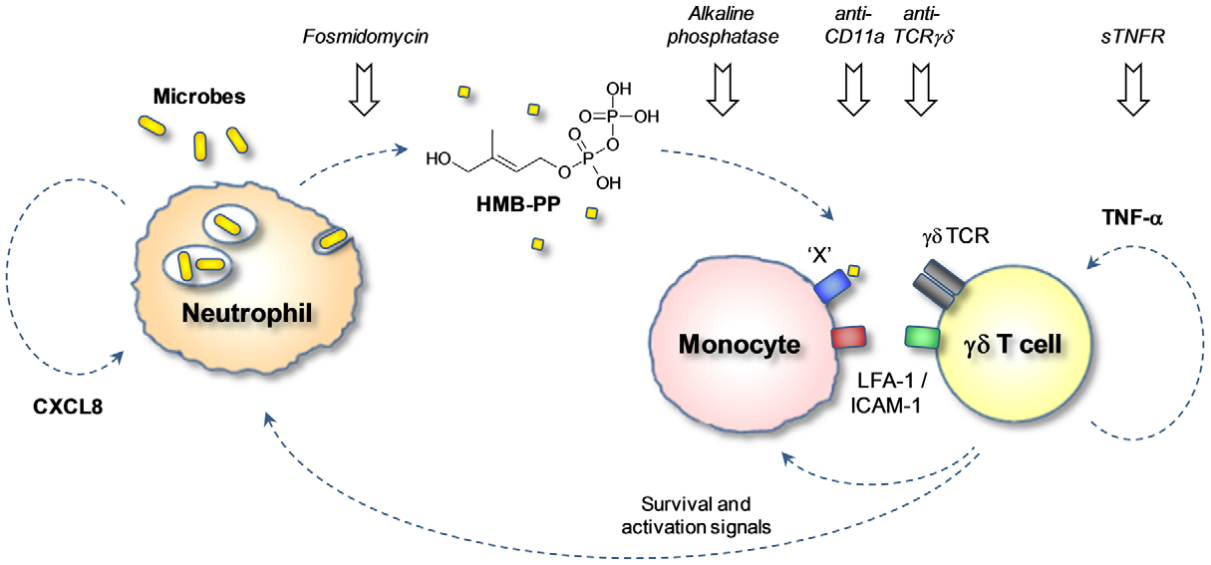
HMB-PP dependent interaction between γδ T cells, neutrophils and monocytes in acute microbial infection. Local secretion of inflammatory chemokines leads to extravasation of neutrophils, monocytes and γδ T cells toward the site of infection. Upon phagocytosis of invading microbes, neutrophils release traces of HMB-PP into the microenvironment where it becomes ‘visible’ to γδ T cells. γδ T cells recognize HMB-PP in the context of a yet unidentified presenting molecule ‘X’ and contact-dependent signals provided by monocytes. Crosstalk between the three different cell types triggers the production of pro-inflammatory cytokines such as TNF-α, which drives local γδ T cell expansion, and chemokines such as CXCL8, which recruits further neutrophils to the site of infection. Activated γδ T cells also provide survival and activation signals such as TNF-α for newly arriving neutrophils and monocytes. This γδ T cell-driven inflammatory reaction can be interrupted at various check-points as demonstrated in the present study.

We next investigated the potential of short-term fosmidomycin treatment to affect γδ T cell activation by inhibiting the bacterial HMB-PP biosynthesis. Prior exposure of bacteria to fosmidomycin for 1 hour did not affect uptake by neutrophils as demonstrated using *Escherichia coli-gfp^+^* (Figure S8B in Text S1), and neither did it affect gross bacterial viability as confirmed by re-plating treated *Enterobacter cloacae* on antibiotic-free plates in order to overcome the competitive inhibition by fosmidomycin (Figure 10A). Yet, pre-incubation of *Escherichia coli*, *Enterobacter cloacae* and *Klebsiella pneumoniae* with fosmidomycin for only 1 hour prior to phagocytosis by neutrophils clearly abrogated their capacity to stimulate Vγ9/Vδ2 T cells. This inhibitory effect on γδ T cell responses was evident both for activation of Vγ9/Vδ2 T cells in triple co-cultures with neutrophils harboring fosmidomycin-treated bacteria (Figure 10A and data not shown) as well as for activation (Figure S8B in Text S1) and proliferation (Figure 10B) of Vγ9/Vδ2 T cells in response to cell-free supernatants from neutrophils harboring fosmidomycin-treated bacteria. Together these results indicate that fosmidomycin not only has a direct antibacterial effect but also possesses immediate anti-inflammatory properties by inhibiting γδ T cell-driven responses (Figure 11), thus making the non-mevalonate pathway an attractive novel drug target for the treatment of acute infection.

## Discussion

Despite its relevance in early infection, the immediate crosstalk of γδ T cells, monocytes and neutrophils in the presence of bacterial pathogens has not been addressed in detail. This is particularly the case in humans who possess a unique γδ T cell population uniformly targeting an invariant non-self-metabolite, HMB-PP. Previous reports already associated the activation of Vγ9/Vδ2 T cells with the production of HMB-PP by microbes. This link was mainly based on the observation that Vγ9/Vδ2 T cell levels are often elevated in the blood of patients infected with HMB-PP producing pathogens [18] and that bacterial extracts prepared from those species activate Vγ9/Vδ2 T cells *in vitro* much better than extracts prepared from HMB-PP deficient micro-organisms [19], [35], [57]. Other investigators have speculated that Vγ9/Vδ2 T cells respond *in vivo* toward infected host cells with dysregulated isoprenoid metabolism leading to accumulation of isopentenyl pyrophosphate (IPP) regardless of the presence or absence of HMB-PP [58]. Here we unequivocally demonstrate that Vγ9/Vδ2 T cells respond to live bacteria upon phagocytosis by neutrophils, that this response is strictly HMB-PP dependent, and that it is amplified by the presence of monocytes providing crucial accessory signals. While it has remained puzzling how the immune system actually ‘sees’ an intracellular metabolite that is unlikely to be secreted or released by live micro-organisms, our findings show that biologically relevant traces of HMB-PP escape into the microenvironment after phagocytosis of extracellular bacteria by neutrophils. These conditions are likely to occur during the acute stage of the infection when Vγ9/Vδ2 T cells and monocytes are co-recruited to the site of inflammation [17] where they encounter neutrophils engaged in clearing invading pathogens (Figure 11).

The present findings explain how HMB-PP may become released at the site of infection. However, the molecular mechanism of HMB-PP recognition by Vγ9/Vδ2 T cells remains poorly understood. Our observation that monocytes were required for Vγ9/Vδ2 T cell responses to phagocytosed bacteria offers important clues. Monocytes and monocyte-derived macrophages or DCs were shown before to provide accessory help and may constitute a pivotal trigger for Vγ9/Vδ2 T cell responses to different bacterial pathogens. In the case of direct infection of monocytic cells, HMB-PP derived from intracellular bacteria may reach the cell surface bound to a presenting molecule [24], [25], [27]. In the case of extracellular bacteria, monocytes may take up or bind soluble HMB-PP released by professional phagocytes and present it to Vγ9/Vδ2 T cells (Figure 11). The HMB-PP presenting pathway remains elusive but may involve cell surface F1-ATPase [59], together with tight cell-cell interactions via LFA-1/ICAM-1 [31], [60], while it is independent of MHC class I, MHC class II, β_2_-microglobulin or CD1 [61]. Of note, any chemical modification of the molecular structure of HMB-PP abrogates its bioactivity by several magnitudes, such that the closely related natural metabolites IPP and DMAPP are >10,000 times less active *in vitro* [39], [40], [62], [63]. This is supported by our previous [35], [64], [65] and present demonstration that HMB-PP deficient bacteria (but which produce IPP and DMAPP) fail to stimulate cytokine production by Vγ9/Vδ2 T cells. Treatment with fosmidomycin or alkaline phosphatase abrogated the Vγ9/Vδ2 T cell responses to HMB-PP producing bacteria and emphasized the importance of HMB-PP for the induction of IFN-γ and TNF-α. However, fosmidomycin or alkaline phosphatase treated cultures as well as cultures involving HMB-PP deficient bacteria did show residual levels of CD69 expression, in line with a role for direct or indirect sensing of microbial TLR ligands [41], [42], [66] that is likely to amplify the overall response. In this respect it is intriguing that our present study identified a crucial role for TNF-α in supporting Vγ9/Vδ2 T cell proliferation, a cytokine which is readily produced not only by activated Vγ9/Vδ2 T cells themselves but also by neutrophils and monocytes exposed to microbial compounds such as LPS. This is in stark contrast to other cytokines produced by innate immune cells such as IFN-α and IFN-β which may induce upregulation of CD69 on Vγ9/Vδ2 T cells but fail to co-stimulate Vγ9/Vδ2 T cell proliferation [32]. Taken together, we identified an inflammatory crosstalk of Vγ9/Vδ2 T cells, neutrophils and monocytes in the presence of HMB-PP producing bacteria that can be manipulated at various check-points: (*i*) the antibiotic fosmidomycin abrogates the microbial HMB-PP production and thus renders bacterial pathogens invisible for Vγ9/Vδ2 T cells; (*ii*) alkaline phosphatase degrades free HMB-PP released by neutrophils into the microenvironment; (*iii*) blocking antibodies against the TCR prevent the recognition of HMB-PP by Vγ9/Vδ2 T cells; (*iv*) blocking antibodies against CD11a disrupt the LFA-1/ICAM-1 dependent contact between γδ T cells and monocytes needed for γδ T cell stimulation; (*v*) and sTNFR neutralizes soluble TNF-α which is released by all three cell types in response to microbial ligands and acts as growth factor for Vγ9/Vδ2 T cells and survival factor for neutrophils (Figure 11).

How does the HMB-PP dependent crosstalk of Vγ9/Vδ2 T cells, monocytes and neutrophils *in vitro* translate into the situation *in vivo* in acutely infected patients? Studies in patients with systemic inflammatory response syndrome suggested a significant role for Vγ9/Vδ2 T cells as early responders after severe insult and identified a correlation between Vγ9/Vδ2 T cell activation and clinical scores [67]. Our own findings in patients with PD-related peritonitis support this notion and demonstrate that the capacity of the causative pathogen to produce HMB-PP and local infiltrates of activated Vγ9/Vδ2 T cells on day 1 are indicative of acute inflammatory responses and may predict the subsequent clinical outcome from infection. It is becoming increasingly clear that the nature of the infection is a major determinant of outcome, and future interventions may well have to focus on subgroups of patients with different forms of infection [68]. A careful re-analysis of peritonitis outcomes from validated registry data [69]–[76] confirms that HMB-PP^+^ bacteria cause clinically more severe infection and emphasizes the need to pay more attention to detailed host-pathogen interactions. Bacterial infection remains a leading cause of morbidity and mortality worldwide, not the least due to the alarming spread of antibiotic-resistant pathogens that is posing an enormous challenge on clinical practice, public healthcare and biomedical research [55], [56]. In most cases, antimicrobial treatment is largely empirical as microbiological culture results are typically not available for 2–3 days. Moreover, many times no organism can be identified, with rates of culture-negative infections occasionally reaching 50% [77]. In contrast, laboratory analyses of immune cells and/or soluble mediators may provide valuable information within a few hours and both aid diagnosis and refine treatment. In this respect, fosmidomycin or related HMB-PP inhibitors might constitute useful combination partners for antibiotic therapy especially in critical cases such as acute peritonitis or sepsis where the intervention has to commence before the nature of the causative pathogen is known, and where activated Vγ9/Vδ2 T cells may contribute to poor clinical outcome. Fosmidomycin targets an essential pathway in a broad range of pathogens [54], [78], [79] and simultaneously abrogates Vγ9/Vδ2 T cell responses [80], [81]. Of note, the inhibitory effect of fosmidomycin on the Vγ9/Vδ2 T cell bioactivity was detectable after only 1 hour of treatment and well below its MIC, *i.e.* under conditions that would easily be achievable in patients. Other ways of specifically manipulating γδ T cell mediated responses may include the use of anti-γδ TCR antibodies or γδ TCR antagonists such as BrH-PCP [82]. Given the importance of TNF-α for γδ T cell proliferation and the association of peritoneal TNF-α levels with morbidity and mortality, one may also advocate the use of TNF-α blocking reagents for the treatment of acute PD-related peritonitis [83].

Taken together our experiments demonstrate that Vγ9/Vδ2 T cells recognize with HMB-PP a small common molecule released by the majority of invading bacteria when they become phagocytosed by neutrophils. Stimulation of Vγ9/Vδ2 T cells at the site of infection is likely to amplify the local inflammatory response with important consequences for pathogen clearance and the development of microbe-specific immunity. However, if triggered at the wrong time or the wrong place, this rapid reaction toward most bacteria may also lead to inflammation-related damage and detrimental clinical outcome. These findings improve our insight into the complex cellular interactions in early infection, identify novel biomarkers of possible diagnostic and predictive value and highlight new avenues for therapeutic intervention.

## Materials and Methods

### Ethics statement

This study was conducted according to the principles expressed in the Declaration of Helsinki and under local ethical guidelines (Bro Taf Health Authority, Wales). The study was approved by the South East Wales Local Ethics Committee under reference number 04WSE04/27. All patients provided written informed consent for the collection of samples and subsequent analysis.

### Patients

The Cardiff study population included 39 adult patients who were receiving PD at the University Hospital of Wales, Cardiff, UK, and were admitted with acute peritonitis between September 2008 and October 2010 (Table S2 in Text S1). Eight stable patients with no infection in the previous 3 months were included in this study as non-infected controls. In addition, microbiological and survival data were obtained from all 739 adult patients who were receiving PD between 1987 and 2008 at the University Hospital of North Staffordshire, Stoke-on-Trent, UK; and from all 2,542 Australian adult patients from the Australia and New Zealand Dialysis Transplant (ANZDATA) Registry who were receiving PD between 2003 and 2008 (Table S2 in Text S1). Diagnosis of acute peritonitis was based on the presence of abdominal pain and cloudy peritoneal effluent with >100 WBC/mm^3^. Infections were grouped into culture-positive and culture-negative episodes, according to the result of the microbiological analysis of the effluent. Bacteria species identified in culture-positive infections were grouped into HMB-PP^+^ and HMB-PP^−^, based on the presence or absence of HMB-PP in the microbial metabolism [17], [19]. Endpoints of outcome analyses were 14^th^ and 90^th^ day mortality and technique failure (catheter removal, transfer to hemodialysis, and/or patient death). In order to rule out a history of previous antibiotic treatment as a potentially confounding factor, outcome studies were restricted to patients with first-episode peritonitis, excluding cases of fungal infection or unrecorded culture results.

### Bacteria culture

Bacteria strains used in this study are listed in Table S1 in Text S1. *Mycobacterium smegmatis* strains were grown aerobically at 37°C in liquid Lemco medium (Oxoid) supplemented with 10 mg/ml peptone, 5 mg/ml NaCl and 0.25% Tween 80 (Sigma), or on solid Lemco plates with 15 mg/ml agar (Fisher). *Listeria innocua* strains were grown aerobically at 37°C in liquid brain heart infusion medium (Oxoid) or on agar plates. *Escherichia coli* laboratory strains and multi-drug resistant clinical isolates of *Acinetobacter baumannii*, *Chryseobacterium indologenes*, *Enterobacter cloacae*, *Enterococcus faecalis*, *Klebsiella pneumoniae*, *Pseudomonas aeruginosa* and *Staphylococcus aureus* were grown in liquid LB broth and on solid Columbia blood agar (Oxoid). Where appropriate, antibiotics were added to the medium: *M. smegmatis-gcpE^+^*, 100 µg/ml hygromycin B; *L. innocua-gcpE^+^*, 7.5 µg/ml chloramphenicol; *E. coli-gfp^+^*, 100 µg/ml ampicillin (all from Sigma). Bacterial susceptibilities to fosmidomycin were determined by microbroth dilution method, according to the Clinical and Laboratory Standards Institute guidelines [84]. A log 2 dilution series of 0.06 to 128 µg/ml allowed the identification of the minimal inhibitory concentration (MIC) where bacterial growth was absent. No defined break points have been acknowledged for fosmidomycin [85], therefore resistance was defined as concentrations >128 µg/ml.

### Cell isolation

Peritoneal cells were harvested from chilled overnight dwell effluents [31], [47]; cell-free supernatants were stored at −70°C. PBMC were isolated from peripheral blood of healthy volunteers using Lymphoprep (Axis-Shield). Vγ9^+^ T cells (>98%) were purified from PBMC using monoclonal antibodies (mAbs) against Vγ9-PE-Cy5 (Immu360; Beckman-Coulter) and anti-PE microbeads (Miltenyi). Monocytes (>98%) were purified using anti-CD14 microbeads (Miltenyi). Neutrophils (>95%) used for bacterial phagocytosis were isolated from peripheral blood using a Lymphoprep gradient followed by dextran sedimentation [86]. Remaining erythrocytes were lysed with ammonium chloride solution (150 mM NH_4_Cl, 10 mM KHCO_3_, 0.1 mM EDTA), and neutrophils were washed in HBSS without Mg^2+^ and Ca^2+^ (Sigma) and resuspended to a final cell concentration of 2×10^6^/ml in HBSS with Mg^2+^ and Ca^2+^ (Sigma) supplemented with 10% human serum. Neutrophils (>98%) used in Vγ9/Vδ2 T cell co-culture experiments were isolated from peripheral blood by initial dextran sedimentation followed by centrifugation through discontinuous Percoll gradients [87]. The cell culture medium used throughout this study was RPMI-1640 with 2 mM L-glutamine, 1% sodium pyruvate, 50 µg/ml penicillin/streptomycin and 10% fetal calf serum (Invitrogen).

### Phagocytosis of live bacteria

Single colonies were grown in culture broth for 18 hours, and bacteria were washed in PBS and resuspended in HBSS with Mg^2+^ and Ca^2+^ supplemented with 10% human serum. Freshly isolated neutrophils were incubated with bacteria at a multiplicity of infection (MOI) of 0.1–100 bacteria per neutrophil for 30–60 min at 37°C, with gentle shaking. In some experiments, bacteria were pre-treated with 0.5–25 µg/ml fosmidomycin for 1 hour prior to phagocytosis. Actual MOIs of all bacterial inocula used were determined by plating out serial dilutions on agar plates and expressed as colony forming units (CFU) per neutrophil. Non-phagocytosed bacteria were washed off three times. For microscopic analyses, neutrophils harboring GFP-expressing bacteria were washed, counterstained with DAPI (Sigma) and fixed in 2% paraformaldehyde. Images were acquired on a Nikon Eclipse 80i fluorescence microscope equipped with a Nikon DXM 1200F camera and processed with Adobe Photoshop. For the generation of cell-free supernatants, neutrophils pre-incubated with bacteria as described above were cultured for 5 hours in complete RPMI-1640. Supernatants were then harvested and cells removed by centrifugation at 12,000 *g* for 10 min. Samples were stored at −20°C and thawed a maximum of 5 times. For some experiments, neutrophil supernatants were treated with 0.015 U/µl shrimp alkaline phosphatase for 30 min at 37°C.

### Triple co-cultures of neutrophils, monocytes and γδ T cells

Unless indicated otherwise, neutrophils were co-cultured for 20 hours in complete RPMI-1640 medium (further supplemented with 8 µg/ml colistin (Sigma) for assays involving multi-drug resistant clinical isolates) with autologous monocytes and γδ T cells at a ratio of 10 neutrophils and 1 monocyte per 1 γδ T cell (10∶1∶1), in the absence or presence of 0.015 U/µl shrimp alkaline phosphatase (Roche). Proliferation assays using γδ T cells that had been pre-labeled with CFSE (Molecular Probes) were incubated for 4–6 days. Controls included co-cultures in the absence or presence of 1–100 nM synthetic HMB-PP [40] or 1–100 ng/ml LPS from *Salmonella abortus equi* (Sigma). In transwell experiments, neutrophils were separated from γδ T cells by 0.4 µm pore polycarbonate membranes (Fisher Scientific). Cell-free supernatants derived from neutrophils after phagocytosis of bacteria were tested in monocyte-γδ T cell co-cultures (1∶1) at a dilution of 1 in 3. Blocking reagents used were anti-IFN-γ (25718; R&D Systems); anti-CD11a (TS1/22) from Dr Ruggero Pardi (DIBIT-Scientific Institute San Raffaele, Milano, Italy); and sTNFR p75-IgG1 fusion protein (Enbrel; Amgen); alone or in combination at 10 µg/ml each. Anti-TCR-Vγ9 (Immu360; Beckman Coulter) was used at 1.25 µg/ml.

### Flow cytometry

Cells were acquired on an eight-color FACSCanto II (BD Biosciences) and analyzed with FloJo 7.6 (TreeStar), using monoclonal antibodies against CD3 (UCHT1), CD15 (HI98), CD25 (M-A251), CD62L (DREG-56), CD69 (FN50), CD86 (2331) and HLA-DR (L243) from BD Biosciences; TCR-Vγ9 (Immu360) and CD40 (mAB89) from Beckman Coulter; and CD11b (ICRF44) and CD14 (61D3) from eBioscience; together with appropriate isotype controls. Cells of interest were gated based on their appearance in side scatter and forward scatter area/height, exclusion of live/dead staining (fixable Aqua; Invitrogen) and surface staining: CD3^−^ CD14^−^ CD15^+^ neutrophils, CD3^−^ CD14^+^ CD15^−^ monocytes, and CD3^+^ Vγ9^+^ γδ T cells. Apoptotic cells were identified using Annexin V (BD Biosciences). For detection of intracellular cytokines, 10 µg/ml brefeldin A (Sigma) was added to cultures 5 hours prior to harvesting. Surface-stained cells were labeled using the Fix & Perm kit (eBioscience) and monoclonal antibodies against IFN-γ (45.15; BD Biosciences) and TNF-α (188; Beckman Coulter).

### Culture supernatants and effluent samples

Soluble cytokines in cell culture supernatants were detected using ELISA kits for IL-1β, IL-6 and IL-17 (R&D Systems); and IFN-γ, TNF-α and CXCL8 (BD Biosciences). All samples were measured in duplicate on a Dynex MRX II reader. Cell-free peritoneal effluents were analyzed on a SECTOR Imager 600 (Meso Scale Discovery) for TNF-α, GM-CSF, IFN-γ, IL-1β, IL-2, IL-6, IL-10, IL-12p70, CXCL8 (IL-8) and soluble IL-6 receptor (sIL-6R). In addition, IL-17, IL-22 and CXCL10 in peritoneal effluents were measured in duplicate on a Dynex MRX II reader, using conventional kits (R&D Systems).

### Statistical analysis

Data were analyzed using two-tailed Student's *t*-tests (GraphPad Prism 4.0), and considered significant as indicated in the figures and tables: ^*^, *p*<0.05; ^**^, *p*<0.01; ^***^, *p*<0.001. Cumulative survival curves as a function of time were generated using the Kaplan-Meier approach and compared using the log rank test (SPSS 16.0).

## Supporting Information

Text S1 Supporting information.
(PDF)
