# Supplementary material for: Human Neutrophil Clearance of Bacterial Pathogens Triggers Anti-Microbial γδ T Cell Responses in Early Infection
Source: PLoS Pathog. 2011 May 12;7(5):e1002040. doi: 10.1371/journal.ppat.1002040 (PMC3093373; doi:10.1371/journal.ppat.1002040)
Supplement: Text S1 — Supporting information. (PDF) [file ppat.1002040.s001.pdf]

Text S1: Supporting Information

Table S1. Bacteria strains used in the present study.

Strain	Relevant feature	Gram	HMB-PP	Fosmidomycin MIC (µg/ml)
Escherichia coli	*[Clontech, Cambridge, UK]*			
E. coli wt	Parental laboratory strain BL21	−	+	*ND*
E. coli-*gfp*$^{+}$	BL21 expressing GFP (pAcGFP)	−	+	*ND*
Mycobacterium smegmatis	*[ref. 34]*			
M. smegmatis wt	Parental laboratory strain mc^{2}155	+	+	*ND*
M. smegmatis-*gfp*$^{+}$	mc^{2}155 expressing GFP (pFLAME7)	+	+	*ND*
M. smegmatis-*gcpE*$^{+}$	mc^{2}155 expressing GcpE from *M. tuberculosis*	+	+++	*ND*
Listeria innocua	*[refs. 35,36]*			
L. innocua wt	Parental laboratory strain APC	+	−	*ND*
L. innocua-*gfp*$^{+}$	APC expressing GFP	+	−	*ND*
L. innocua-*gcpE*$^{+}$	APC expressing GcpE from *Bacillus subtilis*	+	+++	*ND*
L. innocua-*gcpE*$^{+}$/*lytB*$^{+}$	APC expressing GcpE, LytB from *B. subtilis*	+	+	*ND*
**Clinical isolates**	*[this study]*			
Acinetobacter baumannii	Strain 48-694D	−	+	> 128
Chryseobacterium indologenes	Strain S281	−	−	> 128
Enterobacter cloacae ‘A’	Strain 48-12346	−	+	1
Enterobacter cloacae ‘B’	Strain 69-7329	−	+	1
Enterococcus faecalis	Strain IQA 7/09	+	−	> 128
Klebsiella pneumoniae	Strain WCH7	−	+	32
Pseudomonas aeruginosa	Strain 75-3755A	−	+	8
Staphylococcus aureus	Strain S288	+	−	> 128

ND, not determined

Table S2. Characteristics of all patients analyzed in the present study.

	Stable	***Acute peritonitis***		
	**Cardiff (UK)**	**Cardiff (UK)**	**Stoke-on-Trent (UK)**	**ANZDATA (Australia)**
Total number	8	39	385	2,424
Study period	2008-2010	2008-2010	1987-2008	2003-2008
Age (mean ± SD)	61.4 ± 19.5	66.3 ± 11.3	57.0 ± 16.0	58.4 ± 16.7
Women (%)	37.5	35.9	43.1	44.8
Days on PD (mean ± SD)	1088.5 ± 936.2	1106.1 ± 984.9	532.0 ± 581.9	738.7 ± 502.5
14th day mortality (%)	–	2.6	3.6	1.2
14th day technique failure (%)	–	12.5	9.6	13.4
90th day mortality (%)	–	10.3	8.8	1.8
90th day technique failure (%)	–	28.2	19.0	16.8
Culture-positive infections (%)	–	64.1	61.5	85.8
HMB-PP^{+} organisms among positively identified species (%)	–	50.0	26.4	38.6

Table S3. Odds ratios for risk of technique failure and mortality within 90 days after infection, depending on the causative pathogen.

90th day technique failure	**Stoke-on-Trent (UK)**	***p* value**	**ANZDATA (Australia)**	***p* value**	**ANZDATA + Stoke-on-Trent combined**	***p* value**
**Reference: culture-negative**	**1.0**		**1.0**		**1.0**	
HMB-PP⁻	2.0	*	0.8	*n.s.*	1.0	*n.s.*
HMB-PP⁺	7.4	***	3.4	***	4.1	***
**Reference: HMB-PP⁻**	**1.0**		**1.0**		**1.0**	
Gram⁺ HMB-PP⁺	3.1	*n.s.*	2.0	0.066	2.1	*
Gram⁻ HMB-PP⁺	3.8	***	4.3	**	4.0	***

90th day mortality						
**Reference: culture-negative**	**1.0**		**1.0**		**1.0**	
HMB-PP⁻	2.1	*n.s.*	2.3	*n.s.*	1.3	*n.s.*
HMB-PP⁺	6.3	***	5.4	*	2.8	**
**Reference: HMB-PP⁻**	**1.0**		**1.0**		**1.0**	
Gram⁺ HMB-PP⁺	2.9	*n.s.*	2.8	*n.s.*	2.4	*n.s.*
Gram⁻ HMB-PP⁺	3.0	*	2.4	**	2.2	**

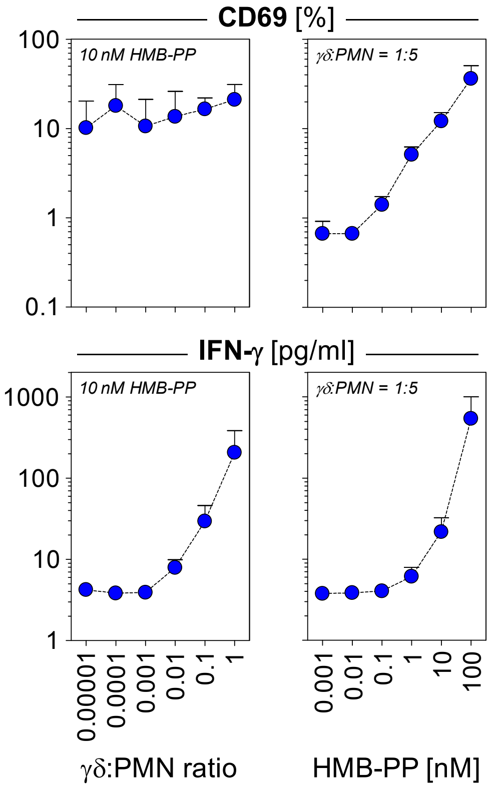

Figure S1. Activation of γδ T cells and dose-dependent secretion of IFN-γ in neutrophil-γδ T cell co-cultures. Expression of CD69 by Vγ9$^+$ CD3$^+$ T cells was determined after 20 hours in culture by flow cytometry; supernatants were analyzed after 20 hours by ELISA. Data shown are mean values + SEM from independent experiments using three different donors.

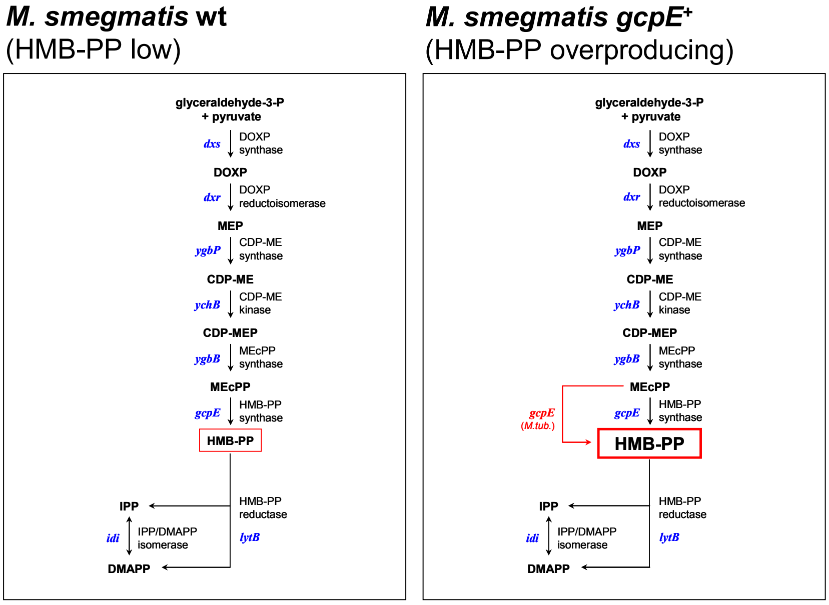

Figure S2. Isoprenoid biosynthesis in the *Mycobacterium* strains used in the present study. *M. smegmatis* possess a fully functional non-mevalonate pathway and hence is capable of producing HMB-PP. Transfection of *M. smegmatis* with an additional copy of *gcpE* from *M. tuberculosis* leads to a 2-3 fold accumulation of HMB-PP compared to the wildtype [35].

Abbreviations: CDP-ME, 4-diphosphocytidyl-2-*C*-methyl-D-erythritol; CDP-MEP, 4-diphosphocytidyl-2-*C*-methyl-D-erythritol 2-phosphate; DMAPP, dimethylallyl pyrophosphate; DOXP, 1-deoxy-D-xylulose 5-phosphate; HMB-PP, (*E*)-4-hydroxy-3-methyl-but-2-enyl pyrophosphate; IPP, isopentenyl pyrophosphate; MEcPP, 2-*C*-methyl-D-erythritol 2,4-cyclopyrophosphate; MEP, 2-*C*-methyl-D-erythritol 4-phosphate.

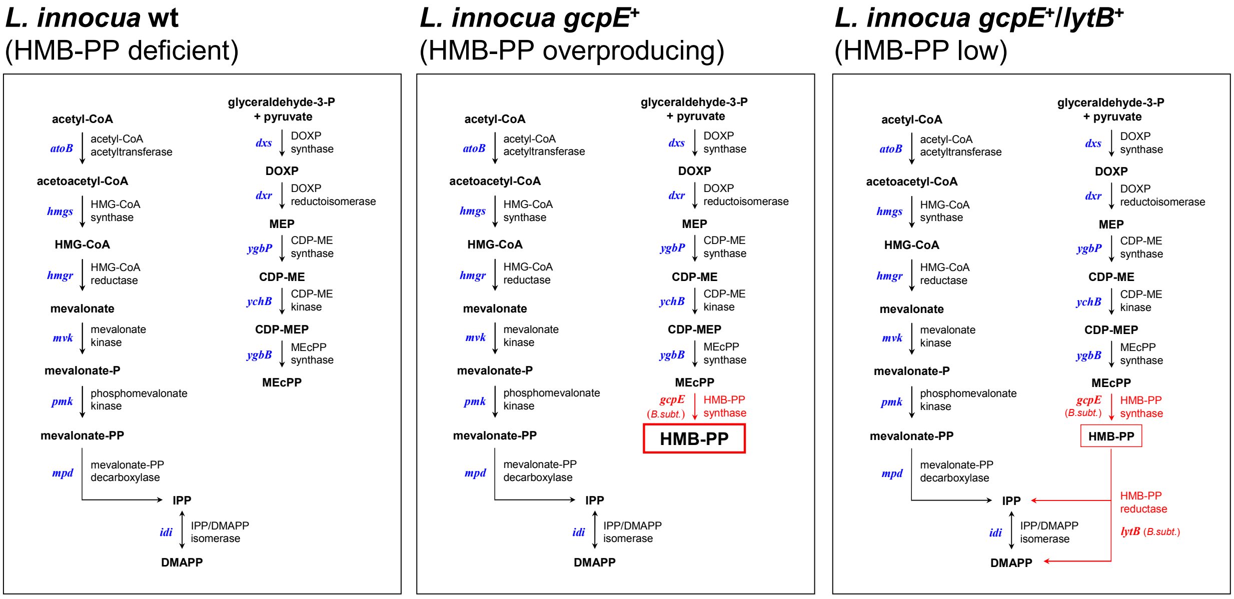

Figure S3. Isoprenoid biosynthesis in the *Listeria* strains used in the present study. *L. innocua* utilizes the mevalonate pathway (left) but also possesses a rudimentary non-mevalonate pathway (right) leading to the production of MEcPP but not of HMB-PP. Transfection of the wildtype strain with a copy of the *gcpE* (*ispG*) gene from *Bacillus subtilis* confers the ability to produce and accumulate HMB-PP; co-transfection of *L. innocua gcpE*$^{+}$ with the *lytB* (*ispH*) gene from *B. subtilis* rescues the non-mevalonate pathway in *L. innocua* and leads to intermediate HMB-PP levels due to reduction of HMB-PP into IPP and DMAPP [36,37].

Abbreviations: CDP-ME, 4-diphosphocytidyl-2-*C*-methyl-D-erythritol; CDP-MEP, 4-diphosphocytidyl-2-*C*-methyl-D-erythritol 2-phosphate; DMAPP, dimethylallyl pyrophosphate; DOXP, 1-deoxy-D-xylulose 5-phosphate; HMB-PP, (*E*)-4-hydroxy-3-methyl-but-2-enyl pyrophosphate; HMG-CoA, 3-hydroxy-3-methylglutaryl-coenzyme A; IPP, isopentenyl pyrophosphate; MEcPP, 2-*C*-methyl-D-erythritol 2,4-cyclopyrophosphate; MEP, 2-*C*-methyl-D-erythritol 4-phosphate; P, phosphate.

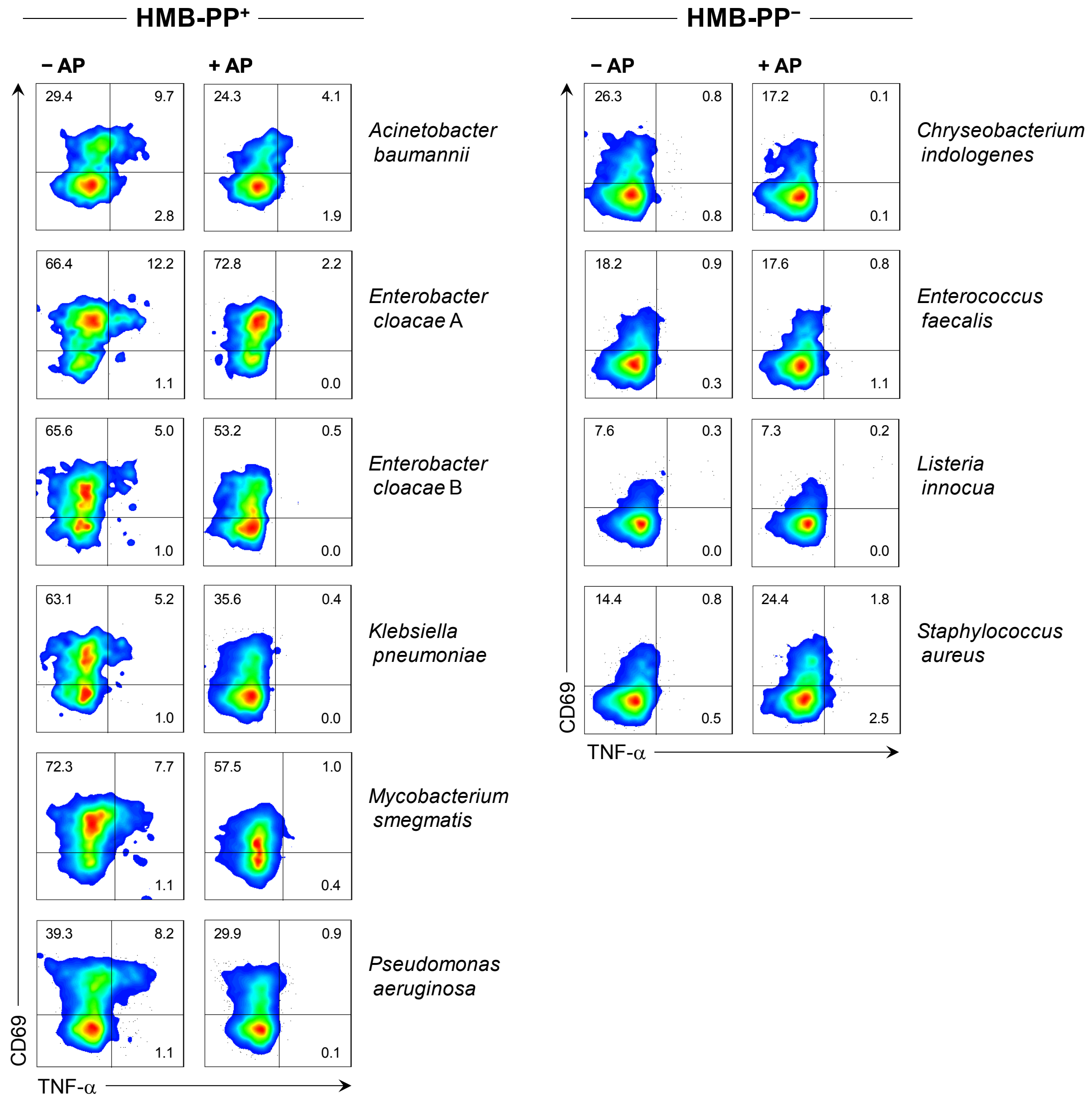

Figure S4. Vγ9/Vδ2 T cells respond to alkaline phosphatase-sensitive molecules released by phagocytosed HMB-PP producing bacteria. Activation of γδ T cells by neutrophils harboring a range of different bacteria: Gram^{-} HMB-PP^{+}, *Acinetobacter baumannii*, *Enterobacter cloacae* (two different strains tested), *Klebsiella pneumoniae* and *Pseudomonas aeruginosa*; Gram^{+} HMB-PP^{+}, *Mycobacterium smegmatis*; Gram^{-} HMB-PP^{-}, *Chryseobacterium indologenes*; and Gram^{+} HMB-PP^{-}, *Enterococcus faecalis*, *Listeria innocua* and *Staphylococcus aureus*; in the presence or absence of alkaline phosphatase (AP). Data shown are representative from independent experiments using 2-5 different donors.

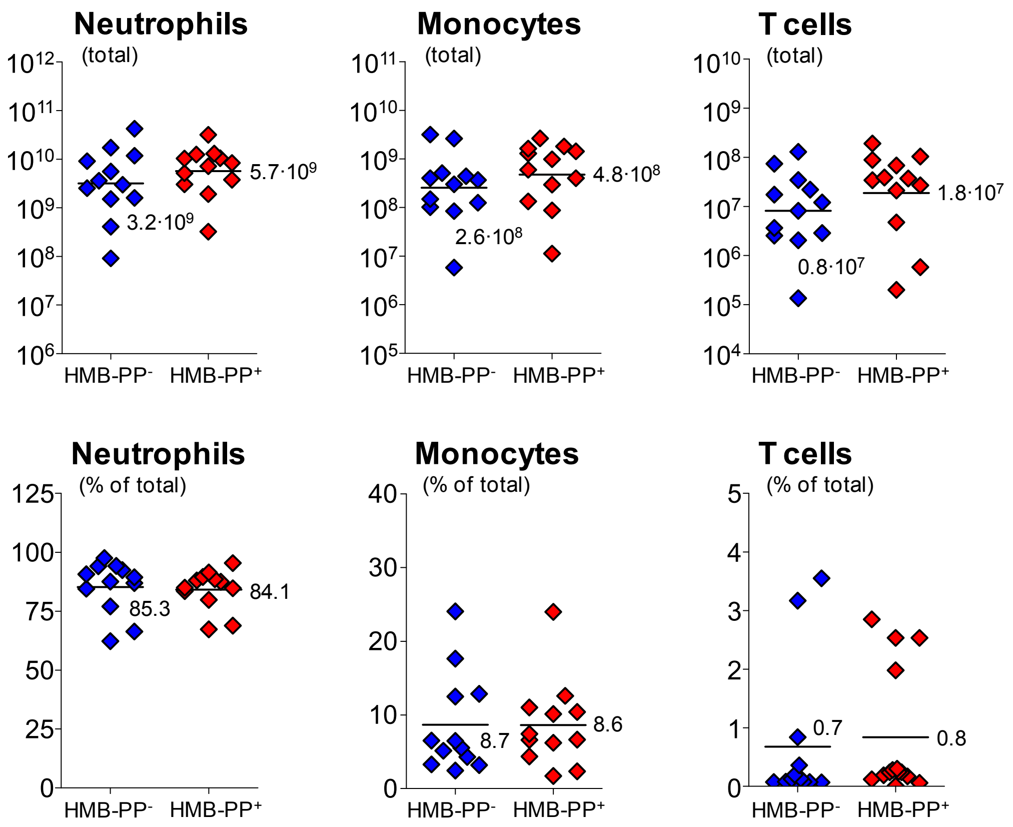

Figure S5. Neutrophil, monocytes and T cells in acute peritonitis are not influenced by the HMB-PP status of the causative pathogen. Total numbers and frequencies of peritoneal CD15$^+$ neutrophils, CD14$^+$ monocytes/macrophages and CD3$^+$ T cells in patients with PD-associated peritonitis on day 1 (the day of hospital admission with a cloudy effluent, *i.e.* before commencement of antibiotic therapy), depending on whether or not the causative pathogen was capable of producing HMB-PP.

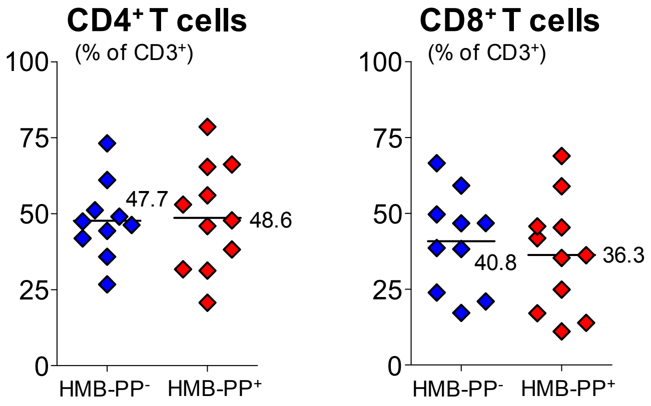

Figure S6. CD4$^+$ and CD8$^+$ T cells in acute peritonitis are not influenced by the HMB-PP status of the causative pathogen. Proportion of CD4$^+$ and CD8$^+$ T cells within all peritoneal CD3$^+$ T cells in patients with PD-associated peritonitis on day 1 (the day of hospital admission with a cloudy effluent, *i.e.* before commencement of antibiotic therapy), depending on whether or not the causative pathogen was capable of producing HMB-PP.

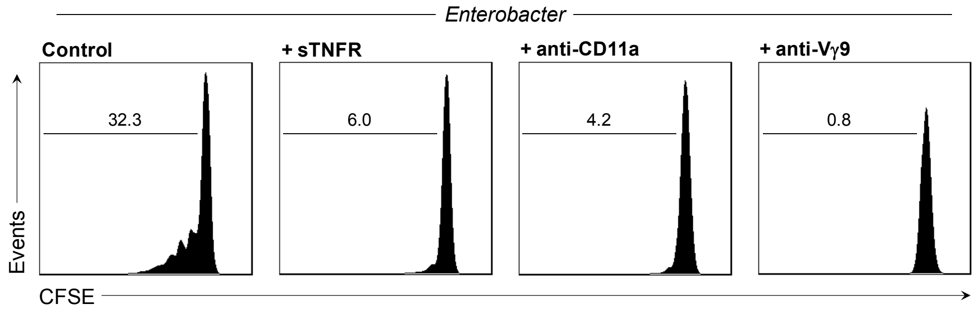

Figure S7. The Vγ9/Vδ2 T cells response to HMB-PP producing bacteria is TCR dependent. Proliferation after 5 days in culture of CFSE-labeled Vγ9/Vδ2 T cells in response to supernatants from neutrophils harboring *Enterobacter cloacae*, in the presence or absence of soluble TNF-α receptor (sTNFR) or antibodies against CD11a or TCR-Vγ9. Data shown are representative of independent experiments using cells from two different donors.

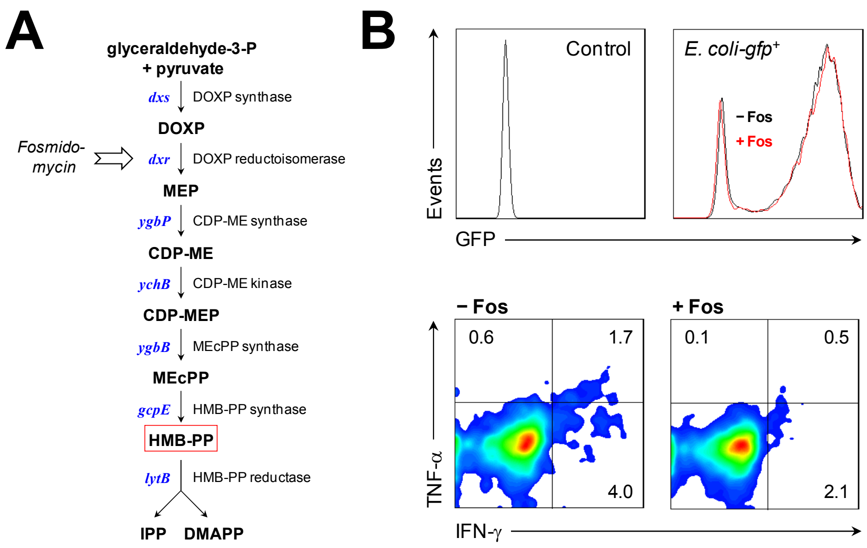

Figure S8. Fosmidomycin abrogates HMB-PP dependent γδ T cell responses to phagocytosed bacteria but does not affect bacterial uptake by neutrophils. (***A***) Overview of the non-mevalonate pathway of isoprenoid biosynthesis. The bacterial genes encoding the enzymes indicated are highlighted in blue. Fosmidomycin blocks HMB-PP production by inhibiting the second step of the pathway. DOXP, 1-deoxy-D-xylulose 5-phosphate; MEP, 2-*C*-methyl-D-erythritol 4-phosphate; CDP-ME, 4-diphosphocytidyl-2-*C*-methyl-D-erythritol; CDP-MEP, 4-diphosphocytidyl-2-*C*-methyl-D-erythritol 2-phosphate; MEcPP, 2-*C*-methyl-D-erythritol 2,4-cyclopyrophosphate; IPP, isopentenyl pyrophosphate; DMAPP, dimethylallyl pyrophosphate. (***B***) Phagocytosis by neutrophils of *E. coli-gfp*$^{+}$ that had been pre-treated or not for 1 hour with 50 μg/ml fosmidomycin (control, neutrophils only). GFP fluorescence was determined 1 hour later. Neutrophil supernatants were collected after 5 hours and added to γδ T cell-monocyte co-cultures for 20 hours. Data shown are representative of independent experiments using cells from two different donors.
